# The C-terminal helix of BubR1 is essential for CENP-E-dependent chromosome alignment

**DOI:** 10.1242/jcs.246025

**Published:** 2020-08-25

**Authors:** Thibault Legal, Daniel Hayward, Agata Gluszek-Kustusz, Elizabeth A. Blackburn, Christos Spanos, Juri Rappsilber, Ulrike Gruneberg, Julie P. I. Welburn

**Affiliations:** 1 Wellcome Trust Centre for Cell Biology, School of Biological Sciences, University of Edinburgh, Edinburgh EH9 3BF, Scotland, UK; 2 Sir William Dunn School of Pathology, University of Oxford, South Parks Road, Oxford OX1 3RE, UK; 3 Chair of Bioanalytics, Institute of Biotechnology, Technische Universität Berlin, Berlin 10623, Germany

**Keywords:** CENP-E, Motor, Kinetochore, Mitosis, Microtubule

## Abstract

During cell division, misaligned chromosomes are captured and aligned by motors before their segregation. The CENP-E motor is recruited to polar unattached kinetochores to facilitate chromosome alignment. The spindle checkpoint protein BubR1 (also known as BUB1B) has been reported as a CENP-E interacting partner, but the extent to which BubR1 contributes to CENP-E localization at kinetochores has remained controversial. Here we define the molecular determinants that specify the interaction between BubR1 and CENP-E. The basic C-terminal helix of BubR1 is necessary but not sufficient for CENP-E interaction, and a minimal key acidic patch on the kinetochore-targeting domain of CENP-E is also essential. We then demonstrate that BubR1 is required for the recruitment of CENP-E to kinetochores to facilitate chromosome alignment. This BubR1–CENP-E axis is critical for alignment of chromosomes that have failed to congress through other pathways and recapitulates the major known function of CENP-E. Overall, our studies define the molecular basis and the function for CENP-E recruitment to BubR1 at kinetochores during mammalian mitosis.

This article has an associated First Person interview with the first author of the paper.

## Abstract (precis)

**Highlighted Article:** An acidic patch in the kinetochore-targeting domain of CENP-E is required for association with the C terminus of BubR1 at kinetochores to facilitate chromosome alignment during mitosis.

## INTRODUCTION

To maintain their genomic integrity, eukaryotic cells must distribute their DNA equally to the daughter cells. Spindle microtubules mediate the segregation of chromosomes by associating with the kinetochore, a large protein complex that mediates the end-on attachment of chromosomes to microtubules. At mitotic onset, chromosomes are dispersed throughout the cytoplasm, posing a challenge for their capture by microtubules from opposite poles, a prerequisite for their accurate segregation. Multiple pathways involving microtubules and motors co-exist to ensure chromosome congression and bi-orientation (Maiato et al., 2017). A subset of chromosomes that lie outside of the interpolar region during spindle assembly are dependent on CENP-E for congression. CENP-E is a large 312 kDa plus-end-directed kinesin that is recruited to unattached and unaligned kinetochores, and to the outer corona, which expands around kinetochores to maximize microtubule capture (Cooke et al., 1997; Yao et al., 1997; Sacristan et al., 2018). Kinetochore-bound CENP-E moves laterally attached chromosomes to the cell equator along microtubules (Wood et al., 1997; Kapoor et al., 2006). CENP-E may also help sort kinetochore-nucleated microtubules and promote end-on attachments and bi-orientation (Shrestha and Draviam, 2013; Sikirzhytski et al., 2018). CENP-E then remains at aligned kinetochores, albeit with lower levels, where it plays a role in maintaining a robust connection between kinetochores and microtubules during metaphase, and during anaphase as the kinetochores are pulled to opposite poles by depolymerizing microtubules (Brown et al., 1996; Vitre et al., 2014).

CENP-E is enriched at unattached and misaligned kinetochores in early mitosis (reviewed in Craske and Welburn, 2020) and is also found at spindle poles (Maffini et al., 2009). The human CENP-E kinetochore-targeting domain has previously been mapped (Chan et al., 1998). Over the years, CENP-E has been reported to interact with multiple kinetochore proteins: BubR1 (also known as BUB1B), CENP-F, Clasp2, Mad1 (MAD1L1), and other interactors such as Septin, CKAP5, NPM1 (Akera et al., 2015; Chan et al., 1998; Maffini et al., 2009; Zhu et al., 2008; Maliga et al., 2013). Post-translational modifications may also enhance CENP-E targeting to kinetochores (Ashar et al., 2000; Zhang et al., 2008). Overall, the molecular basis for CENP-E recruitment to kinetochores remains poorly understood. Kinetochore recruitment of CENP-E has been previously shown to be dependent on the spindle-checkpoint proteins, budding uninhibited by benzimidazole 1 (Bub1) and Bub1-related (BubR1) mitotic checkpoint Ser/Thr kinases; in *Xenopus* and DLD-1 cells, CENP-E kinetochore levels are strongly reduced upon BubR1 depletion (Johnson et al., 2004; Mao et al., 2003). Other studies, however, argue CENP-E levels are not affected by BubR1 depletion (Chan et al., 1999; Ciossani et al., 2018; Kops et al., 2004). CENP-E remains at bi-oriented kinetochores after removal of checkpoint proteins, disassembly of the outer corona and throughout anaphase, indicating CENP-E has multiple as-yet-unidentified binding partners at the kinetochore (Brown et al., 1996; Cooke et al., 1997; Gudimchuk et al., 2013). Here we characterized the kinetochore targeting domain of CENP-E biophysically and used a non-biased approach to find mitotic partners of CENP-E. We found BubR1 as a major interactor and defined the molecular requirements for the BubR1–CENP-E interaction. Overall, we demonstrate BubR1 contributes to CENP-E localization to kinetochores and that this axis is essential for facilitating chromosome alignment.

## RESULTS

**Fig. 1.**
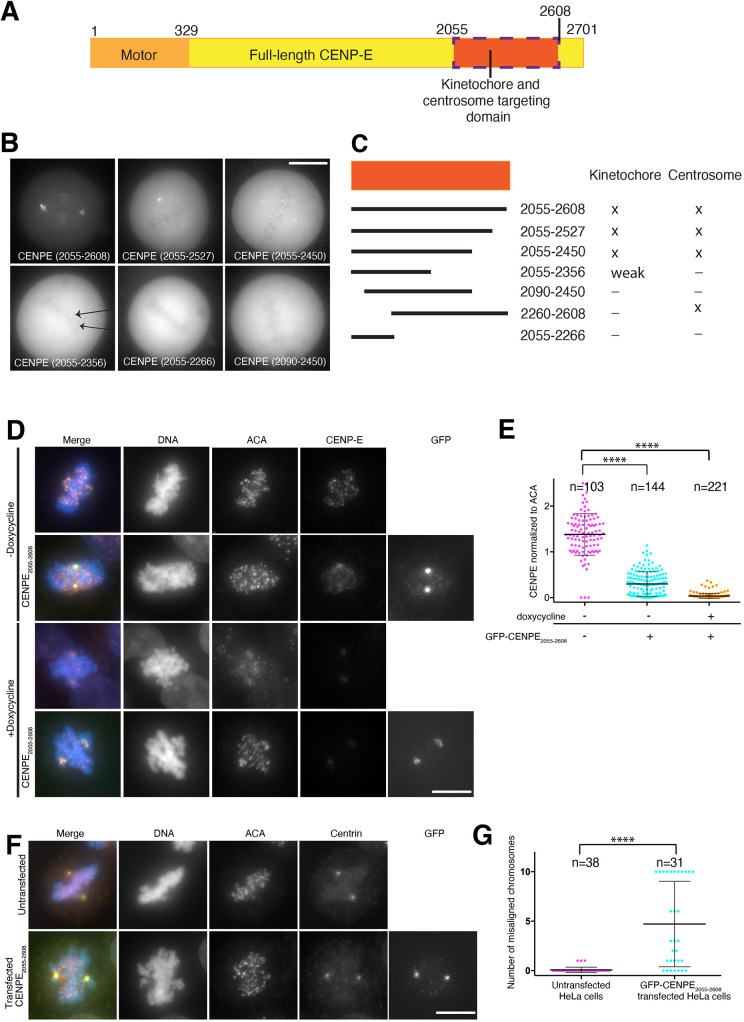
**Mapping of the kinetochore- and centrosome-targeting domain of CENP-E.** (A) Schematic diagram of CENP-E, highlighting the motor and kinetochore- and centrosome-targeting domains. Amino acid residue numbers are indicated. (B) Representative images of live HeLa cells transfected with GFP–CENP-E constructs (arrows indicate kinetochore localization of the construct) and (C) map of the corresponding kinetochore- and centrosome-targeting domains for human CENP-E (× marks the localization of the construct to the kinetochore or centrosome, – marks the absence of localization). Representative images for CENP-E_2260–2608_ are shown in Fig S1C. (D) Representative immunofluorescence images of HeLa cells transfected with GFP–CENP-E_2055–2608_ in the presence (−Doxycycline) and knockout (+Doxycycline) of endogenous CENP-E and stained for endogenous CENP-E, ACA signal and DNA. (E) Scatter plot showing quantification of CENP-E intensity normalized to ACA signal, in the presence and knockout of endogenous CENP-E (doxycycline − and doxycycline +, respectively) and GFP–CENP-E_2055–2608_. Each point represents the intensity of CENP-E over ACA at one kinetochore, with mean±s.d shown. ****P<0.0001 (ordinary one-way ANOVA). (F) Representative immunofluorescence images of wild-type HeLa cells (top) and HeLa cells transfected with GFP–CENP-E_2055–2608_ (bottom) stained for centrin, ACA and DNA. (G) Scatter plot showing the number of misaligned chromosomes in HeLa cells and GFP–CENP- E_2055–2608_-transfected HeLa cells. Each point represents one cell with the corresponding number of misaligned chromosomes, with mean±s.d shown. ****P<0.0001 (unpaired *t*-test). Scale bars: 10 µm.

To define the regulation of CENP-E targeting to kinetochores, we quantitatively examined endogenous CENP-E levels at kinetochores during distinct stages of cell division, CENP-E levels were maximal during prometaphase and decreased during metaphase (Fig. S1A,B). Indeed, CENP-E levels were largely comparable to those in prometaphase upon nocodazole-induced depolymerization of microtubules, creating unattached kinetochores (Fig. S1A,B). To analyse the molecular requirements for CENP-E localization, we precisely mapped the regions of CENP-E isoform 1 that target to kinetochores and centrosomes using transient transfection of CENP-E constructs fused to GFP (Fig. 1A). CENP-E_2055__–__2608_ in the C terminus of CENP-E, largely similar to the previously published kinetochore-targeting construct 1958–2628, was necessary and sufficient for targeting to kinetochores in HeLa cells (Fig. 1B,C) (Chan et al., 1998). The shorter CENP-E_2055–2450_ construct still showed kinetochore localization, whereas an even shorter CENP-E_2055__–__2356_ construct targeted weakly to a subset of kinetochores (Fig. 1B,C). This heterogeneous targeting was previously observed and is likely to reflect different attachment states or kinetochore heterogeneity (Chan et al., 1998). In the absence of the first 35 amino acids in this domain, CENP-E_2090–2450_ lost the ability to target to kinetochores (Fig. 1B,C). However, CENP-E_2260–2608_ localized specifically to a region between the two centrioles or associated closely with one centriole both in interphase and mitosis (Fig. S1C). The intercentriole-interacting proteins remain unknown, and this interaction was not pursued further here.

We then tested whether CENP-E_2055–2608_ dimerizes with endogenous CENP-E at kinetochores. We depleted CENP-E using a Cas9 inducible cell line expressing a CENP-E sgRNA (McKinley and Cheeseman, 2017). CENP-E was largely depleted after 72 h (Fig. 1D,E; +doxycyline). Cells clearly depleted for endogenous CENP-E, as identified by immunofluorescence, were analysed. In the absence of endogenous CENP-E, GFP–CENP-E_2055–2608_ was only weakly targeted to most kinetochores, indicating that CENP-E_2055–2608_ recruitment to kinetochores might depend on the full-length endogenous CENP-E, or that CENP-E removal affects other kinetochore proteins necessary for its recruitment (Fig. 1D; Fig. S1D). However, we observed GFP–CENP-E_2055–2608_ at kinetochores close to spindle poles, suggesting that GFP–CENP-E_2055–2608_ was recruited to these kinetochore subpopulations independently of endogenous CENP-E, through another binding partner (Fig. S1D,E). GFP–CENP-E_2055–2608_ appeared to compete with CENP-E at kinetochores, causing a reduction in endogenous CENP-E at kinetochores (Fig. 1E) in agreement with Schaar et al. (1997). Additionally, CENP-E_2055–2608_ transfection caused many chromosomes to become misaligned (Fig. 1D,F,G), presumably by replacing endogenous motor-domain-containing CENP-E at kinetochores. Overall, these results indicate that CENP-E_2055–2608_ targeting to kinetochores competes endogenous CENP-E.

**Fig. 2.**
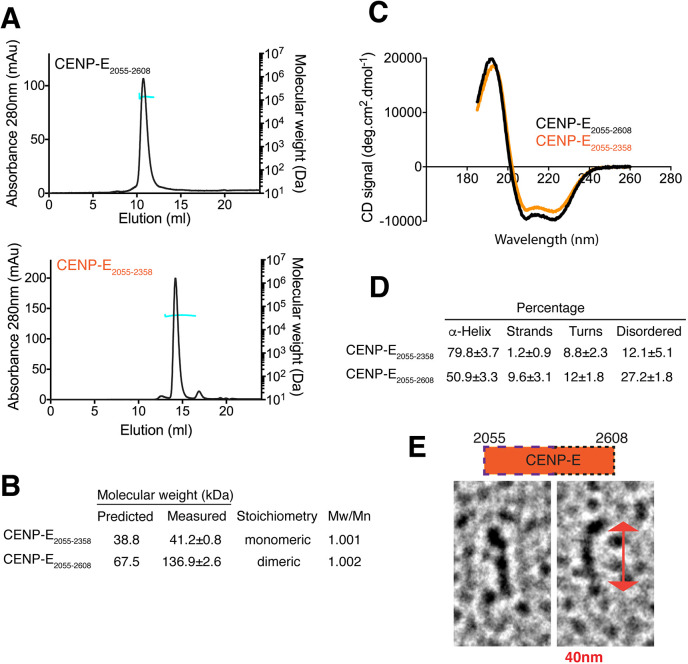
**Biophysical analysis of CENP-E_2055–2608_ and CENP-E_2055–2358_.** (A) Elution profile (black line, left *y*-axis) from a size-exclusion chromatography (SEC) run with subsequent multiangle light scattering (MALS) analysis for CENP-E_2055–2608_ (top) and CENP-E_2055–2358_ (bottom). Outcome of the MALS analysis for the peak is presented in blue (molecular weight, right *y*-axis). (B) Table showing the predicted and measured mass, stoichiometry of the proteins and polydispersity index (Mw/Mn) values. (C) Circular dichroism spectra for 100 µg/ml CENP-E_2055–2358_ (orange) and CENP-E_2055–2608_ (black) indicating that the proteins are predominantly α-helical. (D) Table summarizing the secondary structure features determined from the circular dichroism spectra in C. (E) Representative CENP-E_2055–2608_ particles observed after rotary shadowing. The double arrow shows the length of one particle (40 nm). Diagram shows the kinetochore-targeting domain of CENP-E. The amino acid numbers are indicated. The wide dashed line indicates the first half of the domain that binds to BubR1. The short dashed line indicates the second half of the domain, required for dimerisation.

To define the molecular basis for the CENP-E kinetochore-targeting domain, we expressed and purified recombinant CENP-E_2055–2608_, which robustly targets to both kinetochores and centrosomes, and CENP-E_2055–2358_, which targets weakly to kinetochores. CENP-E_2055–2608_ aggregated in 150 mM NaCl and was maintained in 500 mM NaCl. Size exclusion chromatography–multiangle light scattering (SEC–MALS) analysis revealed that CENP-E_2055–2608_ assembles as a dimer in solution, whereas the minimal kinetochore-targeting domain CENP-E_2055–2358_ was monomeric (Fig. 2A,B). Circular dichroism further defined the secondary structural elements of CENP-E_2055–2608_ and CENP-E_2055–2358_ (Fig. 2C). CENP-E_2055–2608_ has an α-helical content of ∼50.9%, whereas the shorter domain CENP-E_2055–2358_ is 80% α-helical, with 8.8% containing turns and 12.1% containing unstructured regions (Fig. 2D). Rotary shadowing further revealed that CENP-E_2055–2608_ is an elongated domain with a globular region at one end and rod-like shape, supporting a coiled-coil conformation (Fig. 2E). Overall, these data indicated that the CENP-E_2055–2608_ domain is subdivided into an N-terminal monomeric α-helical-rich domain, essential for kinetochore targeting, and a C-terminal domain that provides dimerization properties.

**Fig. 3.**
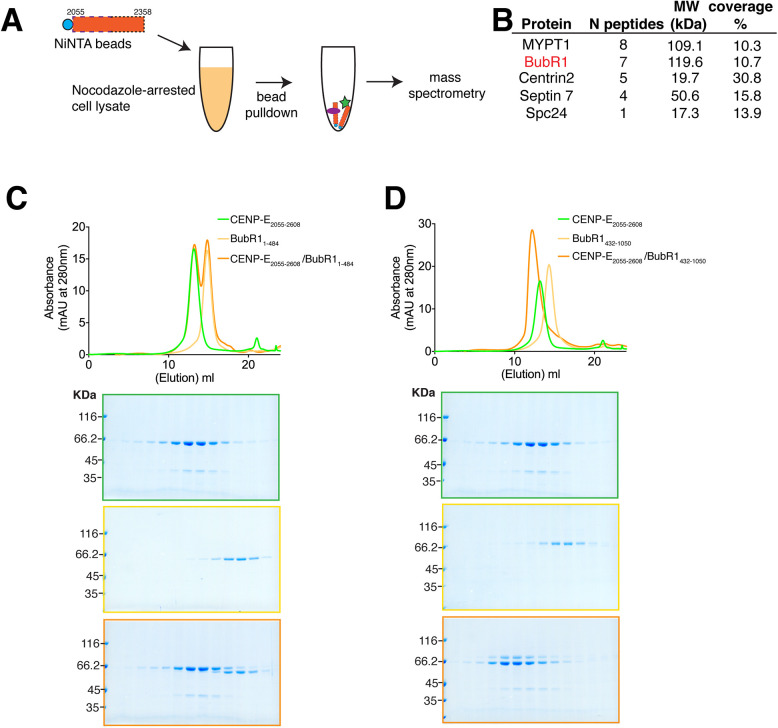
**The CENP-E kinetochore-targeting domain associates with the pseudokinase domain of BubR1.** (A) Schematic showing the identification of CENP- E_2055–2358_-interacting proteins. (B) Mass spectrometry table of proteins identified to co-purify with CENP-E_2055–2358_, reporting the number of peptides identified, molecular weight of protein partners and their percentage peptide coverage. (C,D) Top, SEC analyses and elution profiles for the indicated constructs of CENP-E (green), BubR1 (yellow) and CENP-E–BubR1 (orange). Bottom, Coomassie-stained gels showing elution profiles for the corresponding protein complexes.

We then sought to define the major CENP-E interactors at kinetochores. CENP-E is strongly recruited to unattached kinetochores at mitotic onset (Yen et al., 1991). We incubated CENP-E_2055–2358_ with clarified mitotic cell lysate, from cells arrested using the microtubule-depolymerizing drug nocodazole. We then pulled down CENP-E_2055–2358_ bound to Ni^2+^-NTA beads and associated proteins, which were subjected to mass spectrometry for identification (Fig. 3A; Table S1). We found that CENP-E_2055–2358_ co-purified with BubR1 and MYPT1, a protein phosphatase 1 regulatory subunit 12A (Fig. 3B). To test whether CENP-E directly binds to BubR1, we expressed and purified recombinant BubR1 from insect cells and analysed whether stoichiometric amounts of BubR1 could interact with the longer dimeric kinetochore-targeting domain CENP-E_2055–2608_, using size-exclusion chromatography (SEC). Indeed, full-length BubR1-Bub3 complex interacts with CENP-E_2055–2608_ *in vitro* (data not shown) (Ciossani et al., 2018). We generated two BubR1 constructs containing either the N- or C-terminal domains. The N terminus of BubR1_1–484_ did not co-migrate with CENP-E_2055–2608_ by SEC (Fig. 3C) whereas the C terminus containing the pseudokinase domain, BubR1_432–1050_, did (Fig. 3D), indicating CENP-E_2055–2608_ binds to the pseudokinase domain of BubR1. While we were conducting these experiments, a parallel study also reported an interaction between CENP-E and the pseudokinase domain of BubR1_705–1050_ (Ciossani et al., 2018).

**Fig. 4.**
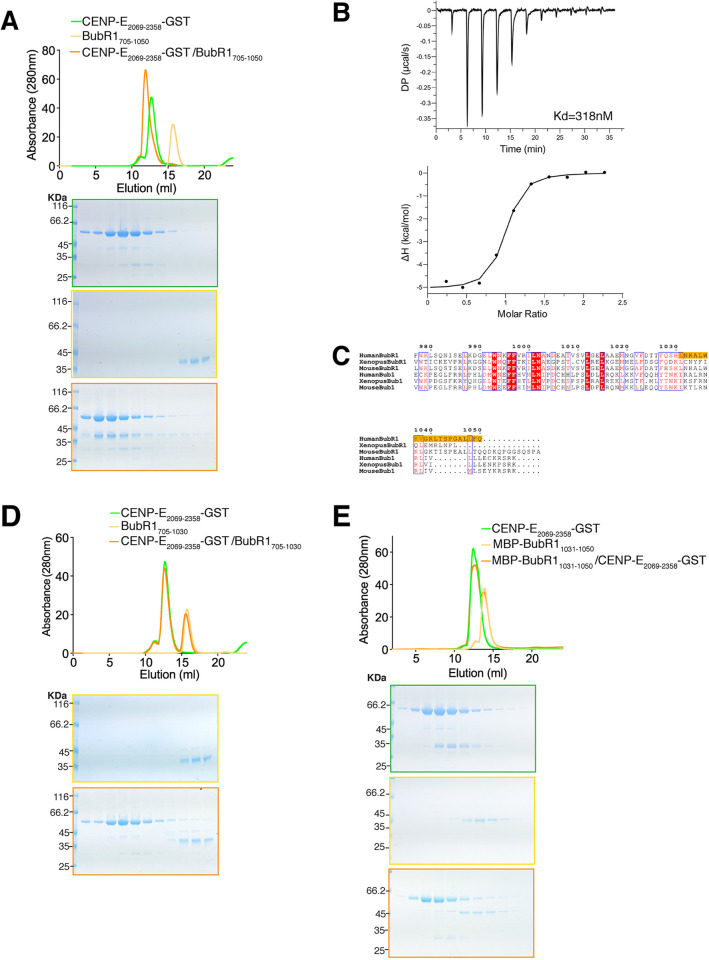
**Requirement of the C-terminal helix of BubR1 for CENP-E binding.** (A) Top, SEC analysis and elution profile for CENP-E_2069–2358_–GST (green), BubR1_705–1050_ (yellow) and CENP-E_2069–2358_–GST–BubR1_705–1050_ (orange). Bottom, Coomassie-stained gels showing elution profiles for the corresponding protein complexes. (B) Thermodynamics of the BubR1_705–1050_–CENP-E_2091–2358_–GST interaction, as determined by isothermal titration calorimetry. The top graph shows the differential power (DP, μcal/s) at each injection (peak). The time of each injection is shown on the *x*-axis. In the bottom graph, the peaks were integrated and are displayed in a Wiseman plot, which shows the enthalpy (ΔH) of each titration on the *y*-axis and the molar ratio on the *x*-axis. The *y*-axis indicates kcal/mol of injectant. The dissociation constant (*K*_d_) between BubR1_705–1050_ and CENP-E_2091–2358_–GST was determined to be 318±90 nM (mean±s.d.). (C) Sequence alignment of the C termini of human, mouse and *Xenopus* Bub1 and BubR1. Boxed red and blue are the conserved and similar amino acids across all six proteins, respectively. Amino acids in red are those with conserved properties. The sequence necessary for BubR1 binding to CENP-E_2055–2608_ is highlighted in orange. (D,E) SEC analysis and elution profile for CENP-E_2069–2358_–GST (green), BubR1_705–1030_ and MBP–BubR1_1031–1050_ (yellow in C and D, respectively), and CENP-E_2069–2358_–GST–BubR1 constructs (orange). Bottom, Coomassie-stained gels showing elution profiles for the corresponding protein complexes.

Our *in vivo* work indicated that CENP-E_2055–2356_ fused to GFP could still weakly associate with kinetochores (Fig. 1B). To test whether post-translational modifications were necessary for this interaction, we tested whether our recombinant construct CENP-E_2055–2358_ interacted with bacterially expressed BubR1_705–1050_ *in vitro*. Indeed, CENP-E_2055–2358_ co-eluted with BubR1_705–1050_, indicating they interact in the absence of post-translational modifications (Fig. S2A). CENP-E_2055–2358_ is monomeric (Fig. 2A,B) and not very stable in low salt concentrations. To stabilize CENP-E_2055–2608_ while mimicking its dimerization, we fused it to a C-terminal GST tag and removed 14 residues at the N terminus. CENP-E_2069–2358_–GST was more stable in low salt concentration and could then be further used to analyse the BubR1–CENP-E interaction by SEC. CENP-E_2069–2358_–GST co-eluted with BubR1_705–1050_, as shown by the shift in the elution profile (Fig. 4A), while GST alone did not (Fig. S2B). The constructs were monodisperse, but we were not able to obtain diffracting crystals of the kinetochore-targeting domain of CENP-E_2055–2358_ alone or bound to BubR1.

To investigate the thermodynamics of the BubR1–CENP-E interaction, we performed isothermal titration calorimetry (ITC). The CENP-E_2069–2358_–GST construct had to be optimized slightly to remove some GST contaminants and degradation. We therefore removed a further 22 residues at the N terminus and purified it in complex with BubR1 before separating the complex in high ionic strength using gel filtration. In this way, we obtained >95% pure BubR1_705–1050_ and CENP-E_2091–2358_–GST. At 37°C the pseudokinase domain of BubR1 bound CENP-E_2091–2358_–GST with mid-nanomolar affinity with a *K*_d_=318±90 nM (mean±s.e.; Fig. 4B). At this temperature, formation of the complex had an exothermic heat signature. The enthalpic and entropic components driving the interaction were of a similar magnitude (Δ*H*=−5.1±0.2 kcal/mol, mean±s.e.; −*T*Δ*S*=−4.1 kcal/mol). The stoichiometry between BubR1 and CENP-E_2091–2358_ was determined to be 1:1 (*N*=0.908±0.016, mean±s.e.). The stoichiometry must be put in the context of full-length dimeric CENP-E. Thus the CENP-E motor is able to bind to two molecules of BubR1.

**Fig. 5.**
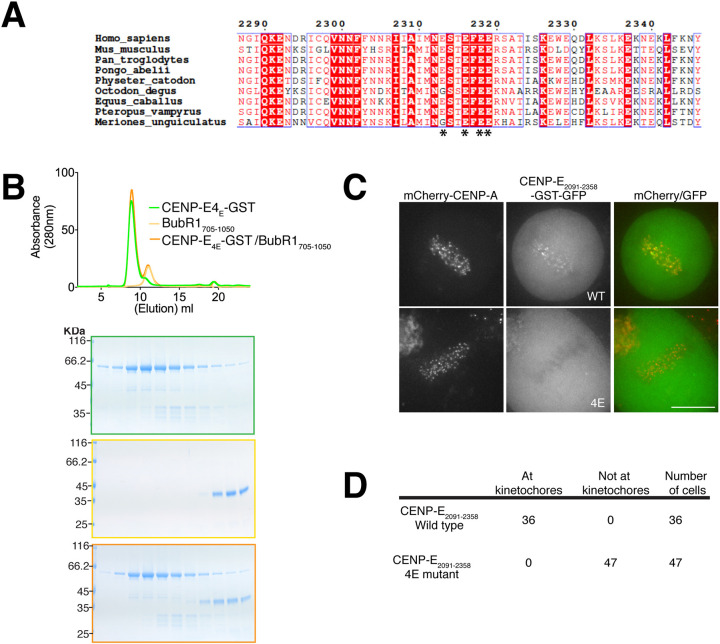
**CENP-E uses an acidic patch to bind BubR1.** (A) Sequence alignment of human CENP-E_2287–2246_ with mouse, chimpanzee, orangutan, sperm whale, degu, horse, flying fox and gerbil CENP-E sequences. Boxed red and blue are the conserved and similar amino acids across all species, respectively. Amino acids in red are those with conserved properties in at least three sequences. The glutamate residues necessary for BubR1 binding in CENP-E are marked with an asterisk (*). (B) Top, SEC analysis and elution profile for CENP-E_4E_–GST (green), BubR1_705–1050_ (yellow) and CENP-E_4E_–GST–BubR1_705–1050_ (orange). Bottom, Coomassie-stained gels showing elution profiles for the corresponding protein complexes. (C) Images of live HeLa cells expressing mCherry–CENP-A, transfected with CENP-E_2091–2358_–GST–GFP (WT) and CENP-E_2091–2358–__4E_–GST–GFP (4E) constructs. Scale bar: 10 µm. (D) Quantification of the targeting to kinetochores for CENP-E_2091–2358_–GST–GFP and CENP-E_2091–2358–4E_–GST–GFP.

We then examined the sequence conservation between Bub1 and BubR1 kinase domains. Previous work highlighted that the C terminus of Bub1 recruits CENP-F to kinetochores (Raaijmakers et al., 2018). We found a predicted helix in the C terminus of BubR1 that showed sequence divergence between human Bub1 and BubR1, but displayed sequence similarity across BubR1 orthologues in vertebrate species (Fig. 4C). We hypothesized this region might be important for the CENP-E–BubR1 interaction Indeed, CENP-E_2069–2358_–GST did not co-elute with BubR1_705–1030_ lacking the last 20 amino acids (Fig. 4D) suggesting that this part of BubR1 is critical for the interaction with CENP-E. However, on its own this basic helix in BubR1_1031–1050_ (pI=10.30) fused to MBP, was not sufficient to interact with CENP-E (Fig. 4E), although we cannot rule out that the MBP would disrupt the interaction. Based on the basic properties of this helix, we also mapped the interaction of BubR1 with CENP-E to the C terminus of CENP-E_2055–2358_. We found a negatively charged region in CENP-E, which we hypothesized could interact with the basic helix of the kinase domain of BubR1. We mutated four highly conserved glutamates (E2313, E2316, E2318 and E2319) to alanines in CENP-E_2069–2358_–GST, named thereafter CENP-E_4E_–GST (Fig. 5A). CENP-E_4E_–GST was co-incubated with BubR1_705–1050_ and analysed by SEC. CENP–E_4E_–GST and BubR1_705–1050_ did not co-elute, indicating that they did not bind to each other (Fig. 5B). In total, our data indicate that the C-terminal helix of BubR1 is necessary but not sufficient to interact with CENP-E_2055–2358_, whereas the glutamate patch (amino acids 2313–2319) in CENP-E is essential to support the interaction.

We then tested whether CENP-E_2091–2358_–GST–GFP could target to kinetochores in cells and whether this recruitment was only dependent on BubR1. Transiently transfected CENP-E_2091–2358_–GST–GFP is dimeric due to the GST tag and is robustly targeted to all kinetochores; however, it did not cause chromosome misalignment (Fig. 5C), unlike CENP-E_2055–2608_. Thus the minimal kinetochore-targeting domain of CENP-E is unlikely to act as a dominant negative at kinetochores. To test whether CENP-E_2091–2358_–GST targeting to kinetochores is specifically dependent on the glutamate patch mediating interaction with BubR1, we generated CENP-E_2091–2358__-4E_–GST–GFP and hypothesized it should not be able to target to kinetochores. Indeed, CENP-E_2091-2358-4E_–GST–GFP was not recruited to kinetochores (Fig. 5C,D). These data indicate that we have identified the minimal kinetochore-targeting region of CENP-E.

**Fig. 6.**
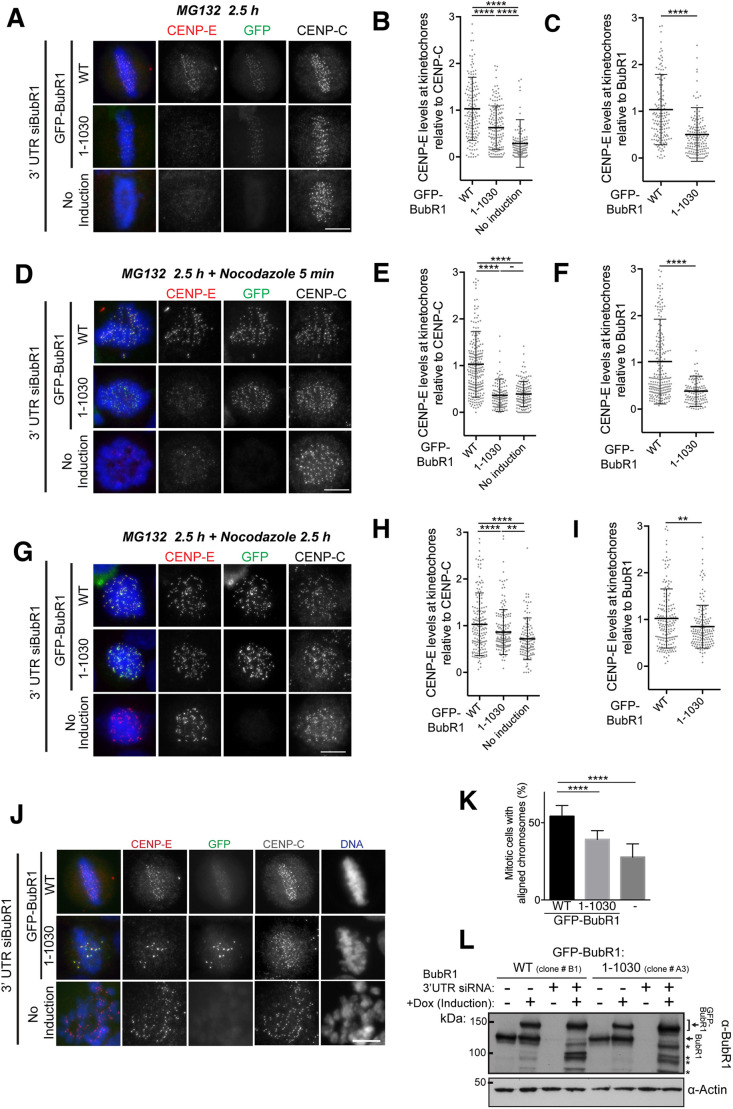
**BubR1 dependency of CENP-E recruitment to kinetochores and chromosome alignment.** (A) Representative immunofluorescence images of HeLa cells treated with BubR1 siRNA and induced to express wild-type GFP–BubR1 (WT) and GFP–BubR1_1–1030_, stained with CENP-E, CENP-C and Hoechst after treatment with MG132 for 2.5 h. No induction: cells with endogenous BubR1 depleted but no induction of the GFP-tagged construct. (B,C) Scatter plots showing CENP-E intensity relative to CENP-C (B) or GFP–BubR1 (C) in cells expressing GFP–BubR1 (WT; *n*=154 kinetochores) or GFP–BubR1_1–1030_ (1–1030; *n*=162 kinetochores), and cells with endogenous BubR1 depleted but no GFP induced (no induction; *n*=135 kinetochores). (D) Same as in A. Cells were treated with MG132 for 2.5 h and nocodazole for 5 min. (E,F) Scatter plots showing CENP-E intensity relative to CENP-C (E) or GFP–BubR1 (F), with cells expressing either GFP–BubR1 (WT; *n*=228 kinetochores) or GFP–BubR1_1–1030_ (1–1030; *n*=133 kinetochores), and cells with endogenous BubR1 depleted but no GFP induced (no induction; *n*=140 kinetochores). (G) Same as in A. Cells were treated with MG132 and nocodazole for 2.5 h. (H,I) Scatter plots showing CENP-E intensity relative to CENP-C (H) or GFP–BubR1 (I), with cells expressing either GFP–BubR1 (WT; *n*=176 kinetochores) or GFP–BubR1_1–1030_ (1–1030; *n*=166 kinetochores), and cells with endogenous BubR1 depleted but no GFP induced (no induction; *n*=131 kinetochores). For A–I, individual kinetochores at the metaphase plate are plotted as grey circles, with mean±s.d. represented by black lines. Ratios are normalized to the mean value of GFP–BubR1 WT. CENP-E:BubR1 ratios were tested using a Student's *t*-test: ****P<0.0001; **P=0.0049; −, *P*>0.05. Measurements were carried out across two independent experiments. (J) Representative immunofluorescence images of HeLa cells treated with BubR1 siRNA and induced to express GFP–BubR1 WT or GFP–BubR1_1–1030_, stained with CENP-E, CENP-C and Hoechst after treatment with MG132 for 2 h. (K) Graph showing percentage of cells with at least one misaligned chromosome for BubR1-depleted cells induced to express GFP–BubR1, GFP–BubR1_1–1030_, or without induction. Data are mean±s.d. Measurements were carried out across two independent experiments. ****P<0.0001 (one-way ANOVA). (L) Western blot for cells in this figure, probed for BubR1 and actin as a loading control. Upper arrow indicates GFP–BubR1 bands, lower arrow indicates endogenous BubR1 band, * indicates GFP–BubR1 degradation products. Scale bars: 10 µm.

We next tested to what extent BubR1 contributes to CENP-E localization at kinetochores. While CENP-E is highly enriched on unattached, spindle checkpoint-active kinetochores, it is still visible on attached, metaphase kinetochores. To evaluate the contribution of BubR1 to CENP-E localization at these distinct kinetochore pools, we used defined synchronization conditions to distinguish the different kinetochore pools. In cells that had been treated with the proteasome inhibitor MG132 for 2.5 h to enrich for attached, spindle checkpoint-silenced kinetochores, both BubR1 and CENP-E were visible at clear, albeit modest levels in control cells (Fig. 6A; Fig. S3A). Cells depleted of BubR1 displayed a near complete loss of CENP-E from kinetochores in this situation, suggesting that CENP-E localization to microtubule-attached kinetochores is dependent on the residual pool of BubR1 retained at metaphase chromosomes (Fig. 6A–C; Fig. S3A,B). This was also the case when Bub1, essential for the recruitment of BubR1 (Johnson et al., 2004), was depleted (Fig. S3A,B). CENP-E localizes both to the outer kinetochore and to the outer corona of chromosomes, which forms preferentially on unattached kinetochores. To test whether the corona proteins are required for CENP-E kinetochore-targeting on attached kinetochores, we depleted the RZZ complex component ZW10, which is involved in corona formation (Fig. S3). ZW10 depletion did not affect CENP-E levels at attached kinetochores under our conditions. These data indicate that, although CENP-E localizes to the outer corona in a RZZ-dependent fashion, CENP-E targeting to kinetochores occurs in the absence of the RZZ complex, as shown previously (Pereira et al., 2018). In the absence of endogenous BubR1, we then expressed full-length BubR1 or BubR1_1–1030_ under an inducible promoter and quantified the corresponding endogenous CENP-E at kinetochores (Fig. 6A–I). At aligned kinetochores, CENP-E levels were reduced by half in the presence of BubR1_1–1030_ (Fig. 6B). CENP-E levels in the presence of BubR1_1–1030_ were, however, higher than those when BubR1 was depleted (Fig. 6A–C), suggesting that BubR1_1–1030_ enables low levels of CENP-E recruitment at kinetochores. Strikingly, when MG132-arrested cells were treated with a short (5 min) pulse of the microtubule-depolymerizing drug nocodazole, a method that has previously been used to test the recruitment of spindle checkpoint components to kinetochores under defined conditions (Vleugel et al., 2015), BubR1-depleted or BubR1_1–1030_-expressing cells were deficient in CENP-E recruitment (Fig. 6D–F). This was in clear contrast to a longer nocodazole treatment (2.5 h), after which the levels of CENP-E observed on BubR1-depleted and BubR1_1–1030_-expressing kinetochores were much more similar (Fig. 6G–I). Taken together, these results suggest that BubR1 primarily facilitates initial CENP-E recruitment to spindle-assembly checkpoint (SAC)-active kinetochores and is not strictly required for CENP-E localization to this subset of kinetochores.

In the absence of BubR1, chromosomes are unable to form stable end-on attachments (Fig. 6J,K) because of the absence of BubR1-recruited PP2A-B56 (Foley et al., 2011; Kruse et al., 2013; Suijkerbuijk et al., 2012). When BubR1_1–1030_ was expressed in the absence of BubR1, most chromosomes were still able to form a metaphase plate, consistent with the idea that PP2A-B56 targeting was restored in this construct. However, in comparison to cells expressing GFP–BubR1_WT_, we observed a significant increase in the percentage of BubR1_1–1030_ cells with misaligned chromosomes (Fig. 6K). In these cells, a small number of chromosomes were unable to congress and displayed high levels of GFP–BubR1_1–1030_ at kinetochores, indicating spindle checkpoint activation (Fig. 6J,K). This phenotype is very similar to that of CENP-E depletion or knockout suggesting that a pool of CENP-E required for efficient chromosome alignment was missing (Fig. 1D) (Schaar et al., 1997). CENP-E, however, was present on the same kinetochores, recruited presumably through a pathway that does not depend on the C terminus of BubR1. Our data so far have demonstrated that the BubR1 C-terminal helix specifically recruits one pool of CENP-E to kinetochores in mitosis and during spindle checkpoint activation. This interaction seems to be required for the productive chromosome alignment and bi-orientation of chromosomes. In the absence of this specific BubR1–CENP-E interaction, the CENP-E recruitment to kinetochores through another pathway does not seem to enable full chromosome alignment. Importantly, in our experiments, the GFP–BubR1 construct was expressed with levels similar to endogenous BubR1 (Fig. 6L). Overall our data indicate that BubR1 recruits CENP-E to bi-oriented chromosomes and is important for rapid recruitment of CENP-E to unattached kinetochores during SAC activation. In the absence of BubR1, another, hitherto uncharacterized pathway, also promotes CENP-E localization to kinetochores during the maintenance of SAC.

## DISCUSSION

CENP-E is an essential motor, targeting to unattached kinetochores and playing a critical role in the congression, maintenance and bi-orientation of chromosomes (Shrestha and Draviam, 2013; Vitre et al., 2014; Wood et al., 1997). Here we show that BubR1 is a nanomolar affinity partner of CENP-E in mitosis and we reveal the molecular basis for the CENP-E–BubR1 interaction. We find that the formation of a CENP-E–BubR1 complex is not dependent on post-translational modifications. Similarly to Ciossani et al. (2018), our work indicates that the pseudokinase domain of BubR1 associates with the C-terminal kinetochore-targeting domain of CENP-E. However, this previous work used a construct that also has the centrosome-targeting domain (Fig. S1C) and a second microtubule-binding site (Ciossani et al., 2018; Gudimchuk et al., 2013). In this study, we map the domain of CENP-E necessary for kinetochore targeting. This domain is monomeric and associates with a 1:1 stoichiometry with BubR1, suggesting that full-length CENP-E can associate with two molecules of BubR1 at one time. Our data reveal BubR1 relies on its divergent and basic C-terminal helix for CENP-E binding, creating a unique and specific association to the mitotic motor. Yet, we find that this helix is not sufficient; on CENP-E a small acidic patch is critical to specify the interaction with BubR1. Mutation of these amino acids prevents the targeting of this CENP-E_2055–2358_ domain to kinetochores.

Previous work on how CENP-E localizes to kinetochores remains unclear, and the extent to which CENP-E requires BubR1 is conflicting. It is likely due to experimental differences between protocols. Indeed, our data suggest that BubR1 primarily facilitates the rapid and initial recruitment of CENP-E to kinetochores at the onset of SAC signaling. Once the SAC is ‘on’ for a significant period of time, we find CENP-E levels become identical at kinetochores in the presence or absence of BubR1, in good agreement with previous work (Ciossani et al., 2018). Our data indicate that BubR1 is a major interactor of CENP-E at kinetochores but there are distinct yet redundant pathways to recruit CENP-E. BubR1 increases the kinetics of CENP-E recruitment to kinetochores during spindle checkpoint activation. The other pathways contribute to a slower but robust targeting of CENP-E to kinetochores. However, they are not sufficient to restore the CENP-E function in chromosome alignment. We therefore suggest that the BubR1-dependent recruitment of CENP-E to kinetochores is particularly important for correct bi-orientation of kinetochores. In addition to aiding its localization, BubR1 might also regulate CENP-E activity at kinetochores, as has been recently suggested (Huang et al., 2019). In the absence of this BubR1-associated pool of CENP-E at kinetochores, the kinetochore-microtubule attachment was compromised, even when CENP-E molecules are recruited via a distinct pathway (Fig. 6J,K). Our work will now further facilitate the identification of BubR1-independent pathways that allow CENP-E to associate with kinetochores, and to define the contribution of this CENP-E pool to chromosome alignment and bi-orientation.

## MATERIALS AND METHODS

### Cloning

To assay the localization in cell culture of CENP-E subdomains, various constructs were generated from CENP-E transcript variant 1 (NM_001813.2) and cloned into pBABE-puro containing an N- or C-terminal GFP tag and using restriction enzymes (Cheeseman and Desai, 2005). Bacterially-expressed constructs were cloned in pET-3aTr (Tan, 2001). pFL MultiBac His-BubR1:Bub3 was a kind gift from Andrea Musacchio (Max Planck Institute of Molecular Physiology, Dortmund, Germany).

### Protein expression, purification and assays

All constructs for bacterial expression were transformed in *E. coli* BL21-CodonPlus (DE3)-RIL (Agilent Technologies). Cultures were induced with 0.5 mM IPTG when OD_600_=0.6 for 4 h at 25°C, or overnight at 18°C for BubR1_705–1050_. Cells were resuspended in lysis buffer (50 mM HEPES pH 7.5, 500 mM NaCl, 40 mM imidazole, 1 mM EDTA and 5 mM β-Mercaptoethanol) supplemented with 1 mM PMSF and cOmplete EDTA-free protease inhibitor cocktail (Roche), and lysed by sonication. The lysate was cleared by centrifugation (50 min, 58,540 ***g***, 22,000 r.p.m.) in a JA 25.50 rotor (Beckman Coulter), filtered and loaded onto a HisTrap HP column (GE Healthcare). Proteins were eluted in elution buffer (lysis buffer with 500 mM imidazole). Constructs containing a 3C protease cleavage site were incubated overnight in dialysis buffer (25 mM HEPES pH 7.5, 300 mM NaCl, 10 mM imidazole, 1 mM EDTA and 5 mM β-Mercaptoethanol) with 3C protease and then loaded onto a HisTrap HP column (GE Healthcare). The protein was then concentrated and loaded on a Superdex 200 Increase 10/300 GL (GE Healthcare) pre-equilibrated in size-exclusion chromatography buffer [20 mM HEPES pH 7.5, 300 mM NaCl (or 500 mM for CENP-E_2055–2608_), 1 mM EDTA and 1 mM dithiothreitol (DTT)].

Constructs for insect cell expression were transfected and expressed in SF9 cells using the Bac-to-Bac^®^ expression system (Thermo Fisher Scientific). Expression was carried out for 72 h at 27°C. Cells were resuspended in lysis buffer supplemented with 1 mM PMSF and cOmplete EDTA-free protease inhibitor cocktail (Roche) and lysed by sonication. The lysate was cleared by centrifugation (60 min, 125,440 ***g***, 40,000 r.p.m.) in a Type 45 Ti rotor (Beckman Coulter), filtered and loaded onto a HisTrap HP column (GE Healthcare). Proteins were eluted and purified by size-exclusion chromatography as for the constructs expressed in bacteria.

Bacteria expressing MBP–BubR1_1031–1050_ were resuspended in MBP lysis buffer (50 mM HEPES pH 7.5, 500 mM NaCl, 1 mM EDTA and 1 mM DTT) supplemented with 1 mM PMSF and cOmplete EDTA-free protease inhibitor cocktail (Roche) and lysed by sonication. The lysate was cleared by centrifugation (50 min, 58,540 ***g***, 22,000 r.p.m.) in a JA 25.50 rotor (Beckman Coulter), filtered and loaded onto an MBPTrap HP column (GE Healthcare). Proteins were eluted in elution buffer (lysis buffer with 10 mM maltose). The fractions containing the protein were concentrated and gel filtered on a Superdex 75 increase 10/300 GL (GE Healthcare) pre-equilibrated in size-exclusion chromatography buffer.

For ITC, CENP-E_2091–2358_–GST was purified in complex with 6×His–BubR1_705–1050_. Both lysates were mixed, cleared by centrifugation, filtered and loaded onto a HisTrap HP column. After overnight incubation with 3C protease, the complex was further purified on a Superdex Increase 200 10/300 GL column (GE Healthcare) in separation buffer (50 mM HEPES pH 7.5, 800 mM NaCl, 1 mM EDTA and 1 mM DTT). The fractions containing CENP-E_2091–2358_–GST and BubR1_705–1050_ were pooled independently then dialysed against the ITC buffer. All binding assays were carried out on a Superdex Increase 200 10/300 GL column in binding buffer (20 mM HEPES pH 7.5, 150 mM NaCl, 1 mM EDTA and 1 mM DTT). Proteins were mixed in equimolar ratio at ∼7 µM.

### Size-exclusion chromatography coupled to multiangle light scattering

Size-exclusion chromatography (ÄKTA PURE**™**, GE Healthcare) coupled to UV, static light scattering and refractive index detection (Viscotec SEC-MALS 20 and Viscotek RI Detector VE3580, Malvern Instruments) were used to determine the absolute molecular mass of the indicated proteins in solution. 100 µl of CENP-E_2055–2608_ and CENP-E_2055–2358_ at 1 mg ml^−1^ were run on a calibrated Superdex-200 10/300 GL Increase (GE Healthcare) size exclusion column pre-equilibrated in gel filtration buffer (described above) at 22°C with a flow rate of 1.0 ml min^−1^. Light scattering, refractive index (RI) and A_280nm_ were analysed using a homo-polymer model (OmniSEC software, v5.02; Malvern Instruments) using the following parameters: ∂A_280nm_/∂c=0.429 AU ml mg^−1^ and 0.530 AU ml mg^−1^ for CENP-E_2055–2608_ and CENP-E_2055–2358_, respectively; ∂n/∂c=0.185 ml g^−1^; and a buffer RI value of 1.336.

### Pulldown with CENP-E_2055–2358_

Cleared mitotic lysate was obtained from 30 confluent 15-cm dishes with HeLa cells arrested in nocodazole (100 ng/ml) for 14 h. Ni-NTA agarose beads (NEB) alone (control) or bound to 600 µg of recombinant CENP-E_2055–2358_–3C cleavage site–6×His were incubated with 1 ml of cleared mitotic lysate for 1 h at 4°C. Beads were spun down and washed in lysis buffer (50 mM HEPES pH 7.5, 1 mM EGTA, 1 mM MgCl_2_, 100 mM KCl, 10% glycerol and 0.05% NP-40) three times. Beads were then incubated with 3C protease at room temperature for 2 h. The supernatant was collected and proteins were precipitated with trichloroacetic acid (TCA) overnight. Precipitated proteins were then washed twice with acetone and proteolytic digestion was carried out with Trypsin (Promega) and Lys-C (Roche) according to the manufacturers' guidelines.

### Mass spectrometry

Following digestion, samples were acidified with 10% trifluoroacetic acid (TFA) until pH<2.5 and spun onto StageTips, as described previously (Rappsilber et al., 2003). Peptides were eluted in 40 μl of 80% acetonitrile in 0.1% TFA and concentrated down to 1 μl by vacuum centrifugation (Concentrator 5301, Eppendorf, UK). Samples were then prepared for liquid chromatography–tandem mass spectrometry (LC-MS/MS) analysis by diluting them to 5 μl with 0.1% TFA. LC-MS/MS analyses were performed on a Q Exactive mass spectrometer (Thermo Fisher Scientific, UK) coupled on-line to an Ultimate 3000 RSLCnano System (Dionex, Thermo Fisher Scientific, UK). Peptides were separated on a 50 cm EASY-Spray column (Thermo Scientific, UK) assembled on an EASY-Spray source (Thermo Scientific, UK) and operated at 50°C. Mobile phase A consisted of 0.1% formic acid in water, while mobile phase B consisted of 80% acetonitrile and 0.1% formic acid. Peptides were loaded onto the column at a flow rate of 0.3 μl min^−1^ and eluted at a flow rate of 0.2 μl min^−1^ according to the following gradient: 2% to 40% mobile phase B in 120 min and then to 95% in 11 min. Fourier transform mass spectra (FTMS) were recorded at 70,000 resolution (scan range 350–1400 *m*/*z*) and the ten most intense peaks with charge between 2 and 6 of the MS scan were selected for fragmentation with an isolation window of 2.0 Thomson for MS2 (filling 1.0×10^6^ ions for MS scan, 5.0×10^4^ ions for MS2, maximum fill time 60 ms, dynamic exclusion for 50 s).

The MaxQuant software platform (Cox and Mann, 2008) version 1.5.2.8 was used to process raw files and the search was conducted against *Homo sapiens* complete/reference proteome set of Uniprot database (released in February, 2016), using the Andromeda search engine (Cox et al., 2011). The first search peptide tolerance was set to 20 p.p.m. while the main search peptide tolerance was set to 4.5 p.p.m. Isotope mass tolerance was set to 2 p.p.m. and maximum charge to 7. A maximum of two missed cleavages were allowed. Carbamidomethylation of cysteine was set as fixed modification. Oxidation of methionine and acetylation of the N terminus were set as variable modifications.

### Circular dichroism

Circular dichroism (CD) spectra in the far-ultraviolet region (185–260 nm) for CENP-E_2055–2358_ and CENP-E_2055–2608_ (0.1 mg/ml) in CD buffer (10 mM potassium phosphate pH 7.5, 200 mM NaF and 0.5 mM DTT) were recorded using a CD spectrometer (Jasco-J-810) at 10°C (1 mm path length quartz cell). Data were analysed using DichroWeb (http://dichroweb.cryst.bbk.ac.uk; Whitmore and Wallace, 2008).

### Isothermal titration calorimetry

Isothermal titration calorimetry (ITC) experiments were carried out to determine the affinity and stoichiometry of the BubR1–CENP-E complex. BubR1_705–1050_ and CENP-E_2091–2358_–GST were extensively dialysed into ITC buffer (20 mM HEPES pH 7.5, 150 mM NaCl, 1 mM EDTA, 0.005% Tween-20 and 0.5 mM TCEP) prior to the experiment to minimize heats of dilution upon titration. Protein concentrations were determined by absorption at 280 nm; extinction coefficients ε for BubR1_705–1050_ and CENP-E­_2091–2358_–GST were 63,370 M^−1^cm^−1^ and 62,800 M^−1^cm^−1^, respectively. 276 μM BubR1_705–1050_ was titrated into 205.4 µl of 25 μM CENP-E­_2091–2358_–GST at 37°C in 11 aliquots: 1 of 0.5 μl followed by 10 of 3.8 μl each. The reference power was set to 3 μcal/s and syringe rotation 750 r.p.m. The enthalpy of binding was analysed with correction for heat of dilution using the software package provided by the instrument manufacturer (Auto-iTC200 microcalorimeter; Malvern Instruments). Data were fitted to a simple binding model with one set of sites. The experimentally derived protein concentrations were fixed and the number of binding sites on CENP-E (*N*), the affinity of the interaction (*K*_d_) and the enthalpy of the interaction (Δ*H*) floated and solved with the Levenberg–Marquardt algorithm after establishing a global minima solution with the Simplex algorithm.

### Low-angle rotary shadowing and electron microscopy

CENP-E_2055–2608_ at a concentration of 100 µg/ml in gel filtration buffer with 30% glycerol were sprayed onto a mica sheet (TAAB). CENP-E_2055–2608_ was shadowed with 2.5 nm of platinum at 5° angle and 9 nm of carbon using a Leica EM ACE600. Replicas were detached in water and placed on non-coated grids (Type 400 mesh, TAAB). Images were obtained using a JEM-1400Plus transmission electron microscope (JEOL) operated at 90 kV. Electron micrographs were acquired using a GATAN OneView camera.

### Cell culture and experiments

HeLa CCL2 cells, from ATCC, were used and maintained in DMEM (Lonza) supplemented with 5% CO_2_ at 37°C in a humidified atmosphere. The inducible HeLa Cas9 sgRNA CENP-E cell line was obtained from Iain Cheeseman (Whitehead Institute, Cambridge, MA, USA; McKinley and Cheeseman, 2017) and maintained in a tetracycline-free medium. Cells were checked monthly for mycoplasma contamination (MycoAlert detection kit, Lonza). Transient transfections were conducted using Effectene reagent (Qiagen) according to the manufacturer's guidelines. GFP–BubR1 wild type (WT) and 1–1030 HeLa cell lines (#B1 and #A3, respectively) were generated with single integrated copies of the desired transgenes using the T-Rex doxycycline-inducible Flp-In system, and were chosen for equal expression levels, as seen by immunofluorescence and western blotting.

GFP–BubR1 was induced 6 h before a 48-h siRNA depletion of endogenous BubR1 using oligonucleotides against the 3′ UTR (5′-GCAATCAAGTCTCACAGAT-3′) (Espert et al., 2014). A second induction was performed 24 h into the siRNA depletion. 26.5 h prior to fixing, cells were subjected to thymidine arrest for 16 h followed by a 10.5 h release. For the final 2.5 h, MG132 was added at 20 µM to increase the metaphase population.

### CRISPR Cas9 knockout

To induce Cas9 expression, cells were treated with 1 µg/ml doxycycline (Sigma Aldrich) for 48–72 h, changing the medium with fresh doxycycline every 24 h to induce the knockout.

### Microscopy

For live-cell imaging, HeLa cells were imaged in Leibovitz L15 medium or DMEM (Life Technologies) supplemented with 10% FBS and penicillin/streptomycin (Gibco) at 37°C using a Deltavision core microscope (Applied Precision) equipped with a CoolSnap HQ2 CCD camera. 4–10 *z*-sections were acquired at 0.5 µm steps using a 60× objective lens. For immunofluorescence, cells were washed with PBS and fixed by one of two methods, either fixed in cold methanol for 10 min at −20°C and then permeabilized with cold acetone for 1 min at −20°C, or pre-extracted with 0.4% Triton-X in PHEM buffer (60 mM Pipes, 25 mM HEPES, 10 mM EGTA, 2 mM MgSO_4_, pH 7.0) for 1 min and then fixed with 3.8% formaldehyde in PHEM buffer for 20 min. For experiments with HeLa Flip-In inducible cells, fixation was performed with PTEMF (20 mM Pipes-KOH, pH 6.8, 0.2% Triton X-100, 1 mM MgCl_2_, 10 mM EGTA and 4% formaldehyde) for 12 min. Immunofluorescence in human cells was conducted using mouse anti-α-tubulin (Sigma; 1:1000), mouse anti-CENP-E (Abcam, Ab5093; 1:1000 or 1:200), rabbit anti-Centrin (kind gift from Iain Cheeseman; 1:1000), guinea pig anti-CENP-C (pAb; MBL PD030; 1:2000) antibodies and human ACA (anti-centromere antibodies; Cambridge Biosciences; 1:100). Hoechst 33342 (Thermo Fisher Scientific; H3570) was used to stain DNA. Nocodazole was used at final concentrations of 0.3 µM for 2.5 h. For the brief nocodazole treatment, a concentration of 3.3 μM was used. A widefield Eclipse Ti2 (Nikon) microscope equipped with a Prime 95B Scientific CMOS camera (Photometrics) was used for imaging. *Z*-sections were acquired at 0.2-µm step size. Images were stored and vizualized using an OMERO.insight client (OME) (Allan et al., 2012). Mean kinetochore fluorescence intensity within a circular region of interest (ROI) with a 10 pixel diameter was measured, with a background intensity recorded in an adjacent cytoplasmic area. Relative CENP-E and BubR1 values for each kinetochore were calculated by subtracting the background values and dividing them by the background corrected ACA or CENP-C signal for that kinetochore. Data was analysed using ImageJ (Schneider et al., 2012).

### Statistics and reproducibility

Statistical analyses were performed using GraphPad Prism 6.0. No statistical method was used to predetermine sample size. All experiments were performed and quantified from at least three independent experiments, unless specified and the representative data are shown.

## Supplementary Material

Supplementary information

Reviewer comments
