# Supplementary material for: The C-terminal helix of BubR1 is essential for CENP-E-dependent chromosome alignment
Source: J Cell Sci. 2020 Aug 25;133(16):jcs246025. doi: 10.1242/jcs.246025 (PMC7473641; doi:10.1242/jcs.246025)
Supplement: Supplementary information [file joces-133-246025-s1.pdf]

Supplementary Figure 1

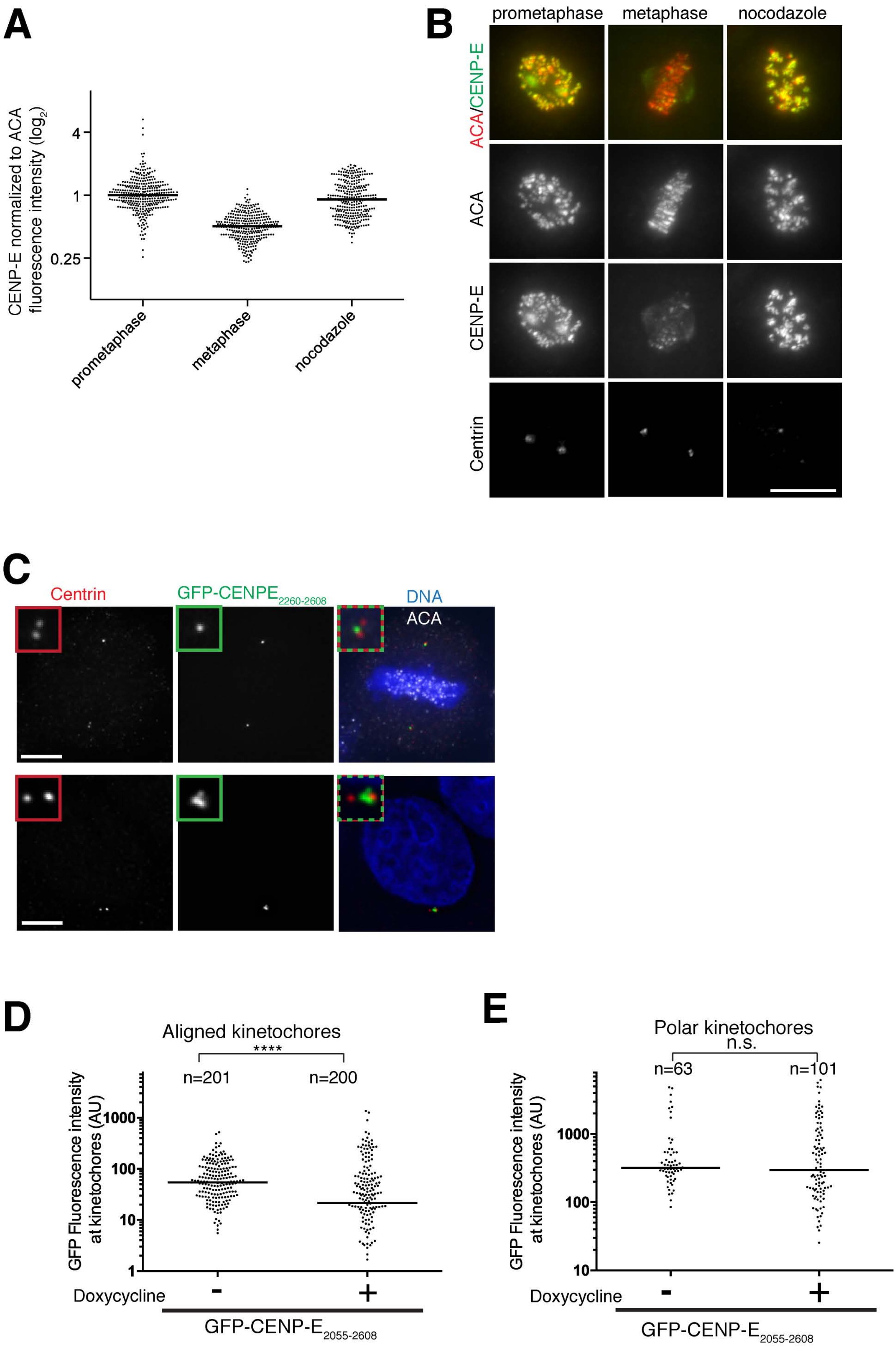

Figure S1: (A) Scatter dot plot showing the quantification of CENP-E intensity normalized to ACA (logarithmic scale) in prometaphase cells, metaphase cells and cells treated with nocodazole. Each point represents the intensity of CENP-E over ACA at one kinetochore. Black line represents the median. (B) Representative immunofluorescence images of HeLa cells for quantification in (A). (C) Representative immunofluorescence images of HeLa cells transfected with GFP-CENP-$E_{2260-2608}$ and stained for kinetochores (ACA), centrioles (centrin2) and DNA (Hoechst). Scale bar: 10 μM. (D, E) Scatter dot plots (logarithmic scale) showing quantification of GFP-CENP-$E_{2055-2608}$ fluorescence intensity at kinetochores in the presence and absence of endogenous CENP-E (knockdown using inducible CRISPR-Cas9) for non polar kinetochores (D) and polar kinetochores (E). Each point represents the intensity of GFP-CENP-$E_{2055-2608}$ at one kinetochore. Black line represents the median. Asterisks indicate ordinary unpaired T-test significance value. ****P<0.0001. Experiments performed three times.

Supplementary Figure 2

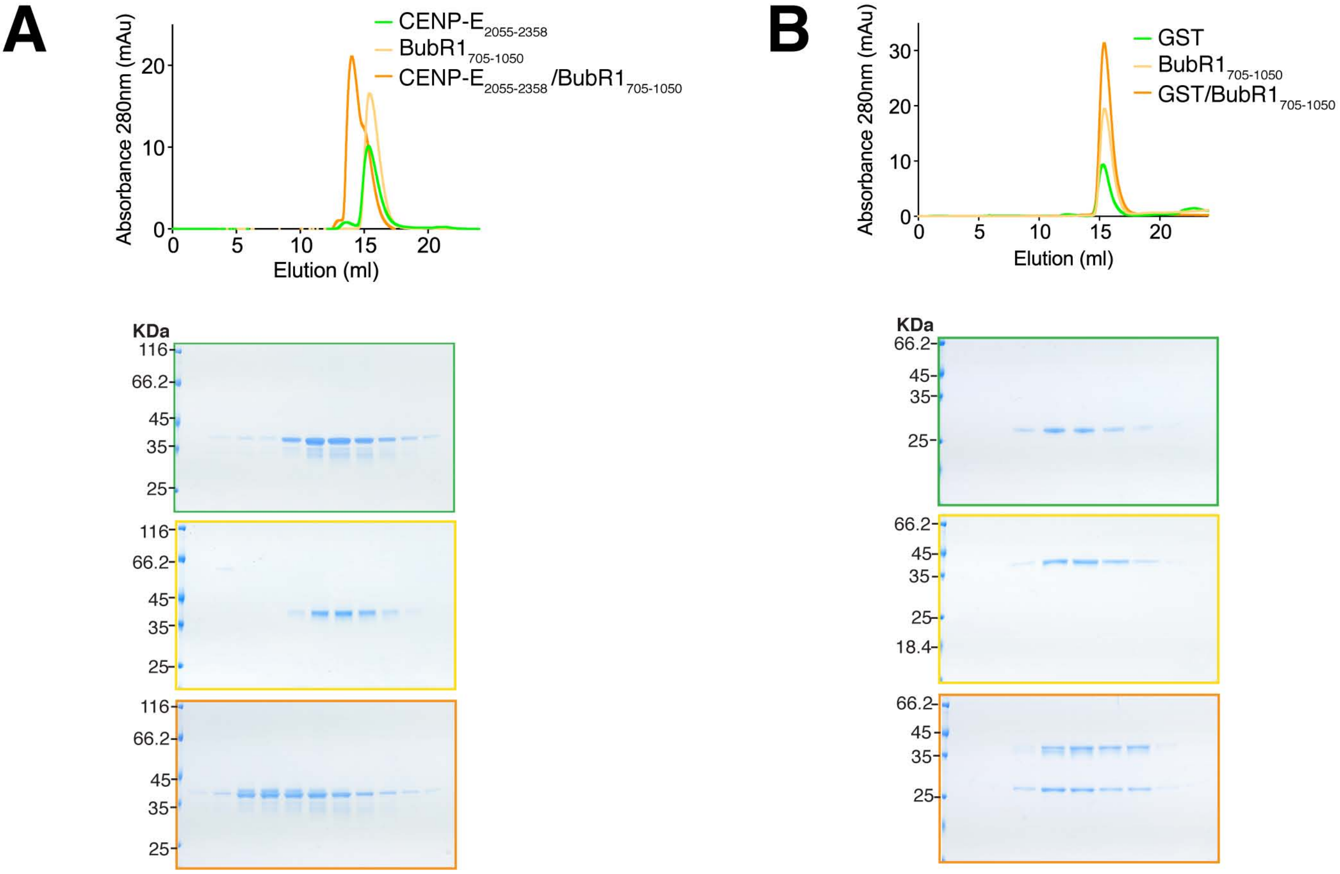

Figure S2: (A) Top, SEC analysis and elution profile for CENP-$E_{2055\text{-}2358}$ (green), $BubR1_{705\text{-}1050}$ (yellow) and CENP-$E_{2055\text{-}2358}$/$BubR1_{705\text{-}1050}$ (orange). Bottom, Coomassie-stained gels showing elution profiles for the corresponding protein complexes. (B) Top, SEC analysis and elution profile for GST (green), $BubR1_{705\text{-}1050}$ (yellow) and GST/$BubR1_{705\text{-}1050}$ (orange). Bottom, Coomassie-stained gels showing elution profiles for the corresponding protein complexes, showing GST does not interact with BubR1.

Journal of Cell Science • Supplementary information

Supplementary Figure 3

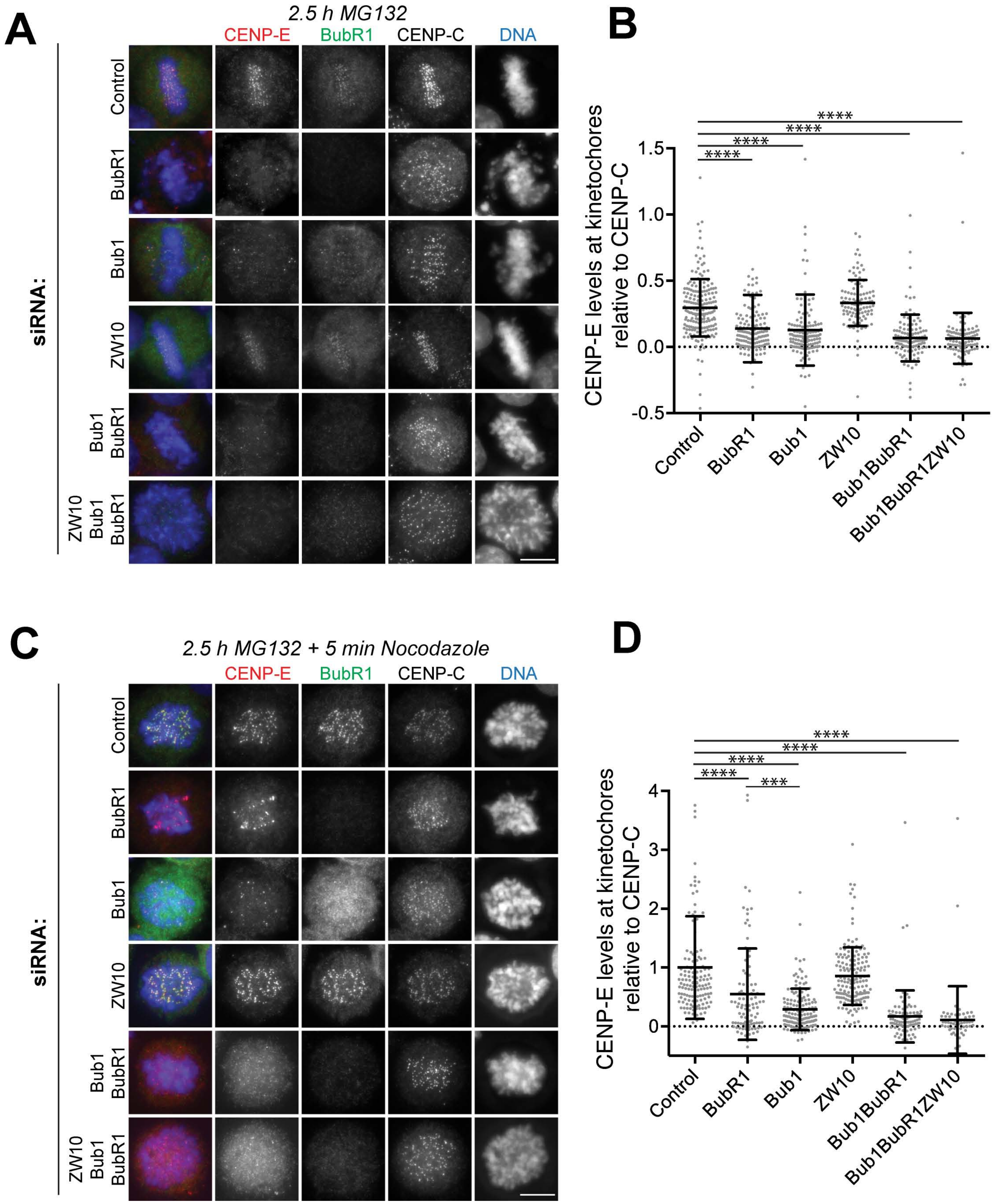

Journal of Cell Science • Supplementary information

Figure S3: (A, C) Representative immunofluorescence images of HeLa cells treated with indicated siRNA and treated with MG132 for 2.5 hours with and without 5 minutes nocodazole treatment. Cells were stained with CENP-E, BubR1, CENP-C and Hoechst. Scale bar: 10 μm. (B, D) Scatter dot plot showing the quantification of CENP-E intensity normalized to CENP-C. Only kinetochores at the metaphase plate were analysed (B). Numbers of kinetochores analyzed for cells treated with 2.5h MG132 and 2.5h MG132+ 5 minutes nocodazole after RNAi depletion were respectively: $n_{control}$= 185, 140; n_{BuBR1}=140, 98; n_{Bub1}=140, 145; n_{ZW10}=111, 159; $n_{Bub1/BubR1}$=120, 100; $n_{Bub1/BubR1/ZW10}$=60, 100. Asterisks indicate significance value performed using a ANOVA one-way test. **** indicate a P-value<0.0001. Experiments performed twice.

Table S1: list of proteins found after CENP- $E_{2055-2358}$ pulldown by mass spectrometry

Click here to Download Table S1
