# Supplementary material for: The C-terminal helix of BubR1 is essential for CENP-E-dependent chromosome alignment
Source: J Cell Sci. 2020 Aug 25;133(16):jcs246025. doi: 10.1242/jcs.246025 (PMC7473641; doi:10.1242/jcs.246025)
Supplement: Reviewer comments [file joces-133-246025_review_history.pdf]

The C-terminal helix of BubR1 is essential for CENP-E-dependent chromosome alignment

Thibault Legal, Daniel Hayward, Agata Gluszek-Kustusz, Elizabeth A. Blackburn, Christos Spanos, Juri Rappsilber, Ulrike Gruneberg and Julie P. I. Welburn
DOI: 10.1242/jcs.246025

Editor: David Glover

Review timeline
Original submission: 9 March 2020
Editorial decision: 7 May 2020
First revision received: 20 May 2020
Editorial decision: 29 June 2020
Second revision received: 29 June 2020
Accepted: 6 July 2020

Original submission

First decision letter

MS ID#: JOCES/2020/246025

MS TITLE: The C-terminal helix of BubR1 is essential for CENP-E-dependent chromosome alignment

AUTHORS: Thibault Legal, Daniel Hayward, Agata Gluszek-Kustusz, Elizabeth Blackburn, Christos Spanos, Juri Rappsilber, Ulrike Gruneberg, and Julie Welburn
ARTICLE TYPE: Short Report

We have now reached a decision on the above manuscript.

To see the reviewers' reports and a copy of this decision letter, please go to: https://submit-jcs.biologists.org and click on the 'Manuscripts with Decisions' queue in the Author Area. (Corresponding author only has access to reviews.)

As you will see, the reviewers raise a number of substantial criticisms that prevent me from accepting the paper at this stage. They suggest, however, that a revised version might prove acceptable, if you can address their concerns. If you think that you can deal satisfactorily with the criticisms on revision, I would be pleased to see a revised manuscript. We would then return it to the reviewers.

We are aware that you may currently be unable to access the lab to undertake experimental revisions. If it would be helpful, we encourage you to contact us to discuss your revision in greater detail. Please send us a point-by-point response indicating where you are able to address concerns raised (either experimentally or by changes to the text) and where you will not be able to do so within the normal timeframe of a revision. We will then provide further guidance. Please also note that we are happy to extend revision timeframes as necessary.

Please ensure that you clearly highlight all changes made in the revised manuscript. Please avoid using 'Tracked changes' in Word files as these are lost in PDF conversion.

© 2020. Published by The Company of Biologists under the terms of the Creative Commons Attribution License (https://creativecommons.org/licenses/by/4.0/).

I should be grateful if you would also provide a point-by-point response detailing how you have dealt with the points raised by the reviewers in the 'Response to Reviewers' box. Please attend to all of the reviewers' comments. If you do not agree with any of their criticisms or suggestions please explain clearly why this is so.

Reviewer 1

Advance summary and potential significance to field

The kinetochore-localized kinesin CENP-E is essential for chromosome congression. Its C-terminal region directly binds the pseudokinase domain of BubR1, yet multiple studies in cultured human cells have reported that BubR1 is dispensable for CENP-E recruitment to kinetochores. Using biochemical biophysical, and cellular assays, Thibault and colleagues reveal molecular details of the BubR1 - CENP-E interaction and provide evidence that BubR1 binding contributes to kinetochore recruitment of CENP-E.

The authors first focus on CENP-E fragment 2055-2608, which has a rod-like appearance in EM micrographs and targets robustly to HeLa cell kinetochores when fused to GFP. Within this CENP-E fragment, residues 2359-2608 are required for dimerization as shown by SEC-MALS. CENP-E(2055-2608) binds BubR1's pseudokinase domain (residues 705-1050) by SEC, and so does CENP-E(2069-2358) when dimerized via fusion to GST. ITC measurements of the interaction using CENP-E(2091-2358)::GST reveals an affinity of about 300 nM. The authors conclude that CENP-E(2091-2358) represents the minimal BubR1 binding fragment. In BubR1, a positively charged C-terminal helical segment (residues 1031-1050) is required but not sufficient for the interaction with CENP-E(2069-2358)::GST. Conversely, point mutations show that an acidic patch in CENP-E located between residues 2313 and 2319 is required for binding of CENP-E(2069-2358)::GST to BubR1 and for kinetochore localization of CENP-E(2091-2358)::GST::GFP.

Finally, the authors show that CENP-E is lost from bi-oriented kinetochores (in cells blocked in metaphase with MG132) when BubR1 (or Bub1) are depleted by RNAi. GFP::BubR1(1-1030) rescues CENP-E localization only partially compared with full-length GFP::BubR1(1-1050), even after a 5-min pulse of nocodazole that generates unattached kinetochores. However, longer treatment with nocodazole mostly rescues CENP-E levels in BubR1-depleted cells, with or without expression of GFP::BubR1(1-1030). The authors conclude that the interaction between CENP-E and BubR1 facilitates rapid initial CENP-E recruitment to unattached kinetochores and is required to maintain CENP-E levels at bi-oriented kinetochores.

The experimental data is of good quality, is clearly presented, and I mostly agree with how it is interpreted. The manuscript makes a valuable contribution to our understanding of how CENP-E is recruited to kinetochores.

Comments for the author

Before publication, the authors should clarify the following points:

- Page 5: "In the absence of endogenous CENPE, GFP-CENP-E(2055-2608) was only weakly targeted to most kinetochores, indicating that CENP-E(2055-2608) recruitment to kinetochores may depend on the full-length endogenous CENP-E or that CENP-E removal affects other kinetochore proteins necessary for its recruitment (Fig 1D, S1D)." The quantification in Figure S1D appears to contradict this conclusion, since kinetochore levels of GFP::CENP-E(2055-2608) are increased in the + dox condition that knocks out CENP-E.

- In Figure 1B and C the authors map the CENP-E region that targets to kinetochores. GFP::CENP-E(2055-2450) localizes, but not when an additional 35 residues are removed from the N-terminus to generate GFP::CENP-E(2090-2450). Yet CENP-E(2091-2358)::GST::GFP localizes robustly to kinetochores, as shown in Figure 4C and D. The authors' explanation for this difference is that CENP-E(2091-2358)::GST::GFP can localize because it is a dimer. Does this, however, not also mean that CENP-E(2091-2358)::GST::GFP might tolerate further N-terminal truncations beyond residue 2091?

© 2020. Published by The Company of Biologists under the terms of the Creative Commons Attribution License (https://creativecommons.org/licenses/by/4.0/).

This question is important, because the authors refer to CENP-E(2091-2358) as the minimal kinetochore-targeting domain when the minimal domain may in fact be considerably shorter especially since the acidic patch that is required for kinetochore targeting is located near the C-terminus of this fragment. Absent additional experimental evidence, the authors should tone down their conclusion with respect to having “precisely” (page 10) defined the minimal kinetochore targeting domain of CENP-E.

- Page 5: based on the CD measurements in Figure S2C and D, the authors conclude that “the region 2358-2608 responsible for dimerization…is highly likely an alpha-helical coiled coil.” This may well be the case, but I don't quite understand how the authors conclude this from the CD measurements: 2055-2358 has a significantly higher percentage of alpha-helices than 2055-2608, so does this not imply that 2359-2608 must contain a large percentage of non-helical segments?

- Mutations in the CENP-E acidic patch (E2313A, E2316A, E2318A, and E2319A) prevent kinetochore localization of CENP-E(2091-2358)::GST::GFP. Is there a specific reason why the authors did not characterize this interesting mutant in the context of full-length CENP-E? Since the acidic patch mutant prevents binding to BUBR1, it would be a precise tool to assess the functional significance of this interaction in cells and would have nicely complemented the BubR1(1-1030) mutant in Figure 4. Related to this, the authors conclude on page 10: “On CENP-E a small acidic patch is critical to specify the interaction with BubR1. Mutation of these amino acids prevents the targeting of this CENP-E(2055-2358) domain to kinetochores and consequently compromises chromosome alignment.” The conclusion about chromosome alignment is not valid because this would have to be assessed in the context of full-length mutated CENP-E.

- As shown in Figure 4, GFP::BubR1(1-1030) results in misaligned chromosomes with high levels of CENP-E. The authors conclude that “The BubR1-dependent recruitment of CENP-E to kinetochores is therefore essential for correct biorientation of kinetochores.” (page 10). Why does it have to be related to recruitment per se? I am not disputing the conclusion that BubR1 facilitates initial kinetochore recruitment of CENP-E, but the authors should also consider the possibility that the effect of the GFP::BubR1(1-1030) mutant on chromosome congression may reflect a role of the BubR1 interaction in regulating CENP-E activity.

Other minor issues/suggestions:

- Figures in general: in many instances (e.g. Figure 1D and F), the CENP-E residue numbers are too small to read comfortably. I would recommend not using subscript for residue numbers or increasing font size.

- Figure 1D: it would be good to show some blow ups of the polar kinetochores for GFP::CENP-E(2055-2608).

- Figure 2C: it might be informative to include additional Bub1 orthologs in the aligment to show the extent to which this region is divergent between BubR1 and Bub1.

- Figure 4: it would be helpful to indicate in the figure panels that BubR1 is depleted by RNAi.

- Figure S3B: in addition to the number of peptides the % sequence coverage should also be shown.

- Page 3: “Kinetochore-bound CENP-E moves laterally attached chromosomes to the cell equator along microtubules (Wood et al., 1997). It would be appropriate to also cite Kapoor et al (2006), PMID 16424343.

- Page 6, repetitive sentence: “To stabilize it while mimicking dimeric CENP-E(2055-2608), we fused it to a C-terminal GST and removed 14 residues at the N terminus, to stabilize it while mimicking dimeric CENP-E(2055-2608).”

- Page 7: “We found a longer loop in the C terminus of BubR1…”, but then later “on its own this basic helix in BubR1…”. Is it a loop or a helix?

© 2020. Published by The Company of Biologists under the terms of the Creative Commons Attribution License (https://creativecommons.org/licenses/by/4.0/).

- Page 9: “After a longer nocodazole treatment (60 minutes),…(Fig 4G-I)”. In Figure 4, the nocodazole treatment is said to be 2.5 h.

Reviewer 2

Advance summary and potential significance to field

Efficient chromosome congression is paramount for robust mitosis. Legal et al. aimed to assess the contribution of the interaction between BubR1 spindle assembly checkpoint protein and CENP-E kinesin, in chromosome congression. The authors first generated CENP-E truncated proteins to define the minimal motif needed for kinetochore targeting in human cells. They next used these fragments to test if CENP-E interacts with BubR1 in vivo. This was followed by a methodical biochemical analysis of BubR1-CENP-E interaction in vitro that led to the identification of essential interaction motifs in both proteins. Finally, the authors used truncated BubR1 proteins unable to bind CENP-E to show that BubR1-dependent recruitment of CENP-E contributes to efficient chromosome congression. In general, the data presented in this article are of high quality and are well documented in the text. With a few exceptions the line of thought is easy to follow, the methods are exhaustingly described and figure legends contain all the necessary information. Overall, this study provides mechanistic insight into chromosome congression, but a few points require (mostly textual) revision or toning down. In particular, the conclusion that BubR1 is required for “initial” CENP-E recruitment should be toned down, and biochemical analyses and immunofluorescence quantification need clarification.

Comments for the author

Major points:

1. What was the control for the MS analysis? How did the authors ensure that the interaction between CENP-E 2055-2358 and BubR1 or MYRT1 is specific? Can we be sure that this interaction occurs at kinetochores? CENP-E 2055-2358 appears to also bind CEP170 (17 peptides), Cep55 (14 peptides) and Aurora-A (12 peptides). One would expect these interactions to occur at centrosomes, where the CENP-E 2055-2358 fragment does not bind (Fig. 1 B and C).

2. Fig. 2E - The text should specify that this fragment was fused with an MBP tag that is significantly larger than 20aa of BubR1. A proper control for this experiment would be the MBP-BubR1-705-1050 fragment, which should co-elute with the CENP-E 2055-2356 fragment. Such a control would exclude the possibility that the MBP tag perturbs the interaction. Otherwise it is difficult to conclude that this basic helix region is "not sufficient" for binding to CENP-E.

3. “We then tested whether CENP-E2091-2358-GST-GFP could target to kinetochores in cells and whether this recruitment was only dependent on BubR1” (p. 7) - It will be helpful to motivate the use of the 2091-2356 fragment and not the 2055-2356 in this experiment. The 2091-2356 fragment was used specifically to assess thermodynamics of BubR1/CENP-E interaction (Fig. 2B) but was otherwise not tested in any other assay. Why not use the 2055-2356 fragment that the authors showed to bind to BubR1 in vivo (Table S1 of MS analysis) and in vitro (Fig. 2A)? Does adding GST require elimination of 22 residues from the N-terminus for in vivo kinetochore localization? If yes, this should be noted in the text.

4. “These data indicate that while CENP-E can localize to the outer corona, the RZZ complex is not a CENP-E recruiting-factor to kinetochores, as shown previously (Pereira et al., 2018)” (p. 8) - As the authors stated, the fibrous corona is most prominent on unattached kinetochores. Therefore, it is not surprising that depletion of corona components did not change CENP-E levels at attached kinetochores. This does not exclude that corona components could contribute to the prometaphase CENP-E recruitment. To conclude that RZZ complex does not contribute to CENP-E recruitment, the authors should show that CENP-E levels are still high at unattached kinetochores under ZW10 depletion. It is also not clear from the context if the authors corroborate or contradict previous findings by Pereira et al., 2018.

© 2020. Published by The Company of Biologists under the terms of the Creative Commons Attribution License (https://creativecommons.org/licenses/by/4.0/).

5. Fig. 4A-I. - One would expect that unattached kinetochores harbored more CENP-E, but the quantitation does not show an increase in the CENP-E/CENP-C ratio. If the ratios were normalized relative to WT in each experiment, then this should be explained in the legend. Along these lines CENP-E levels should go up at unattached kinetochores created by brief nocodazole treatment. Therefore, it is likely that CENP-E levels went up in both BubR1 WT and "no induction" (due to residual BubR1 left), while the BubR1 1-1030 fragment failed to recruit CENP-E to the newly formed unattached kinetochores. This is consistent with the authors' idea that BubR1 is important for initial CENP-E recruitment. However, with 2.5 h nocodazole treatment, it looks like CENP-E levels are higher with the 1030 fragment compared to no induction. The authors stated that CENP-E levels are equal in these conditions, but the statistics represented in Fig. 4H do not test these two conditions. In fact, it appears that BubR1 1-1030 expressing cells are more similar to BubR1 WT than to "no induction". This result seems inconsistent with the conclusions that BubR1 "is not strictly required for CENP-E localization" (p. 9) and that "another pathway also promotes CENP-E localization to kinetochore in the absence of BubR1" (p. 9).

6. Fig, 4A-I - The figure reads 2.5h = 150 minutes nocodazole, not 60 minutes as described in the text. Was nocodazole added together with MG132? If yes, then one would assume that these kinetochores were likely never attached unlike the kinetochores measured in Fig. A-C. This might contribute to the different phenotype observed in Fig. 4D-F and Fig. 4G-I, since kinetochore composition and biochemical state change throughout mitosis. While nocodazole treated cells lose attachments and recruit SAC components, it is unclear if artificial destabilization of fully mature attachments (MG132) can be compared with initial CENP-E recruitment at kinetochores that have never been attached. One example of this is PP1 phosphatase that lingers at kinetochores even after 30min of high nocodazole treatment (DeLuca et al., 2011 J Cell Sci). Also, what happens to the fibrous corona that was most likely gone in MG132 treated cells? Is it reformed? A better way to assess the requirement of BubR1 for initial CENP-E recruitment would be to quantify CENP-E in prometaphase cells when the initial attachments take place. Unless these points can be addressed, the statement that the "BubR1 facilitates initial CENP-E recruitment to SAC-active kinetochores" (p.9) should be toned down.

7. Fig. 4L - There are multiple bands on the gel in the "induced" lanes. Are these degradation products? Since these are well-defined bands, they may indicate stable truncations fragments bearing BubR1 epitope. The authors should indicate which bands correspond to the expected BubR1 protein/fragment. Also, one can see a faint band of BubR1 size in BubR1 1-1030 and BubR1 WT cells under BubR1 depletion. It is then possible that the increased level of CENP-E in BubR1 1-1030 mutant as compared to "No induction" condition (Fig. 4A-B) is due to either higher level of the endogenous BubR1 or to the presence of some truncation products. A blot for GFP could help show which bands are the induced proteins as opposed to endogenous BubR1.

8. "In the absence of this CENP-E pool at kinetochores, the kinetochore-microtubule attachment is compromised, even when high levels of CENP-E are present (Fig 4J, K)" (p. 10). - How did the authors define "high levels" of CENP-E in Fig. 4J? It is possible that the few unaligned chromosomes in BubR1 1-1030 harbor more CENP-E than "no induction" cells, but less CENP-E than prometaphase BubR1 WT cells prior to biorientation. This comparison is needed to justify the phrase "even when high levels of CENP-E are present".

Minor points:
1. Introduction - It would be useful for the readership to cite the recent experimental evidence that kinetochore expansion maximizes capture via fibrous corona (Sacristan et al., 2018 NCB).

2. Introduction p.2 – "CENP-E is enriched at unattached and misaligned kinetochores in early mitosis". Although this may be a common observation in the mitotic field, it is important for the broader cell biology readership to cite previous work showing this.

3. Introduction - Was centrosome localization of the endogenous CENP-E reported previously? If yes, a citation of the relevant work is needed here.

4. "Indeed full-length BubR1 interacts with CENP-E2055-2608 in vitro (data not shown)." (p.4) – Why is this data not shown?

© 2020. Published by The Company of Biologists under the terms of the Creative Commons Attribution License (https://creativecommons.org/licenses/by/4.0/).

5. Fig. 1B and C - What was the reason for looking at CENP-E localization in metaphase? The primary function of CENP-E is chromosome congression and its highest kinetochore localization is observed in early mitosis, therefore one might miss kinetochore-localization domains used prior to congression. As the authors pointed out, CENP-E localization changes throughout mitosis, presumably due to the composition of the fibrous corona and SAC proteins. Therefore, it would be informative to compare the behavior of given fragment in early vs late mitosis. A protein fragment in its localization at kinetochores throughout mitosis would be a great candidate for a SAC-independent localization.

6. Fig. 1B and C - 2260-2608 fragment is not depicted.

7. Fig. S1D and E – “ However, GFP-CENP-E2055-2608 was strongly enriched at kinetochores close to spindle poles, suggesting GFP-CENP-E2055-2608 was recruited to these kinetochore subpopulations independently of endogenous CENP-E, through another binding partner (Fig 1D, S1E)” (p. 2). Bringing both graphs to the same scale would help assess this statement. Currently the median value for polar chromosomes is unreadable. Is there a significant difference between conditions tested in S1E graph? If so, suggesting that GFP-CENP-E recruitment at polar chromosomes is independent on the endogenous protein, is most likely incorrect.

8. “Overall these results indicate CENP-E2055-2608 targeting to kinetochores outcompetes endogenous CENP-E” (p. 2). - "Outcompetes" implies some kind of direct measurement of competition between the endogenous and exogenous CENP-E. However, we do not know the ratio of these proteins in the cytoplasm. Based solely on decreased immunofluorescent signal, it would be safer to simply say "... competes with endogenous CENP-E".

9. “To stabilize it while mimicking dimeric CENP-E2055-2608, we fused it to a C- terminal GST and removed 14 residues at the N terminus, to stabilize it while mimicking dimeric CENP-E2055-2608” (p. 5) – there is a repetition in this sentence.

10. Table S1 - One of the highest scoring proteins is CENP-E itself. Did the authors detect any peptides belonging to the endogenous CENP-E? Absence of such peptides would reinforce the finding that this protein region lacks dimerization capacity in vivo.

11. Isothermal titration calorimetry (ITC) - This paragraph would benefit from additional explanation (method section). How was the stoichiometry determined to be 1:1 and what led to the conclusion that CENP-E motor is able to bind two molecules of BubR1?

12. “We then examined the sequence conservation between Bub1 and BubR1 kinase domains.” - It is not clear from the text why authors are performing such comparison. Bub1 peptides (3) were found in the MS analysis using CENP-E 2055-2356 fragment (Table S1). Therefore, why would one expect that the longer loop in the C-terminus of BubR1 absent in Bub1 be responsible for specific CENP-E 2055-2356 and BubR1 interaction? This line of reasoning requires additional explanation.

13. “Indeed, CENP-E2069-2358-GST did not co-elute with BubR1705-1030 lacking…” – Do the authors mean 2055-2356 fragment? As a general comment, the authors should ensure that all the fragment names in the text match the names in the figures.

14. “Since BubR1 mediates the kinetochore localization of only one pool of CENP-E (see below), this suggests…” (p. 8) - It is challenging to follow the reasoning based on the result that was not yet presented. Alternatively the authors could specify which figure are they referring to when saying "below". Up to this moment in the text, the authors did not provide any evidence that BubR1 mediates recruitment of any CENP-E pool to kinetochores. We only know that BubR1 and CENP-E interact, possibly at kinetochores (see Major comment 1).

15. Fig. S3C - What was the reason for not using the CENP-E 2055-2358 fragment that is sufficient to bind BubR1 according to the MS analysis?

16. “To evaluate BubR1’s contribution to CENP-E localization (…) Fig. 4A, S4A” (p.9) – Figure S5A should be referred to here.

© 2020. Published by The Company of Biologists under the terms of the Creative Commons Attribution License (https://creativecommons.org/licenses/by/4.0/).

17. Fig. 4A-K – Images and graphs should be annotated as "siBubR1" in all conditions. This would make it easier to read the figure without digging into the figure legend.

18. Fig. 4 C, F, I - were the CENP-E levels compared to BubR1-GFP signal or to the total BubR1? It is important to make this distinction because siRNA never depletes 100% of the endogenous protein.

19. Consistent with the minor comment #20 – Why are CENP-E/ACA intensity ratios in Fig. 1S reported as medians but CENP-E/CENP-C ratios in Fig. 4 as means?

20. Fig. S4A - The authors showed that CENP-E 2055-2608 from bacteria and BubR1 432-1050 from insect cells interact in vitro. What is the reason behind using a different BubR1 fragment (705-1050) in the analysis represented in Fig. S4A? This fragment lacks 273aa as compared to the previously tested BubR1 432-1050.

21. Fig. S5A - It should be explicitly mentioned in the figure legend that only metaphase plate kinetochores were counted, since Bub1 depletion generates some unaligned kinetochores.

22. Methods section, Microscopy - There is no mentioning of Aurora-B or Aurora-A inhibition in this paper but the names and concentrations of their inhibitors (ZM1 and MLN) are listed.

First revision

<u>Author response to reviewers' comments</u>

Reviewer 1 Comments for the Author:

- Page 5: "In the absence of endogenous CENPE, GFP-CENP-E(2055-2608) was only weakly targeted to most kinetochores, indicating that CENP-E(2055-2608) recruitment to kinetochores may depend on the full-length endogenous CENP-E or that CENP-E removal affects other kinetochore proteins necessary for its recruitment (Fig 1D, S1D)." The quantification in Figure S1D appears to contradict this conclusion, since kinetochore levels of GFP::CENP-E(2055-2608) are increased in the + dox condition that knocks out CENP-E.
We apologize for this mistake. The signs had been swapped over. The figure has now been corrected.

- In Figure 1B and C the authors map the CENP-E region that targets to kinetochores. GFP::CENP-E(2055-2450) localizes, but not when an additional 35 residues are removed from the N-terminus to generate GFP::CENP-E(2090-2450). Yet CENP-E(2091-2358)::GST::GFP localizes robustly to kinetochores, as shown in Figure 4C and D. The authors' explanation for this difference is that CENP-E(2091-2358)::GST::GFP can localize because it is a dimer. Does this, however, not also mean that CENP-E(2091-2358)::GST::GFP might tolerate further N-terminal truncations beyond residue 2091? This question is important, because the authors refer to CENP-E(2091-2358) as the minimal kinetochore-targeting domain when the minimal domain may in fact be considerably shorter, especially since the acidic patch that is required for kinetochore targeting is located near the C-terminus of this fragment. Absent additional experimental evidence, the authors should tone down their conclusion with respect to having "precisely" (page 10) defined the minimal kinetochore targeting domain of CENP-E.
We have indeed not tested shorter constructs than the one starting at 2090 as a dimer. We have now removed "precisely" from our sentence.

- Page 5: based on the CD measurements in Figure S2C and D, the authors conclude that "the region 2358-2608 responsible for dimerization…is highly likely an alpha-helical coiled coil." This may well be the case, but I don't quite understand how the authors conclude this from the CD measurements: 2055-2358 has a significantly higher percentage of alpha-helices than 2055-2608, so does this not imply that 2359-2608 must contain a large percentage of non-helical segments?

© 2020. Published by The Company of Biologists under the terms of the Creative Commons Attribution License (https://creativecommons.org/licenses/by/4.0/).

We agree with the reviewers and we have now removed this sentence.

- Mutations in the CENP-E acidic patch (E2313A, E2316A, E2318A, and E2319A) prevent kinetochore localization of CENP-E(2091-2358)::GST::GFP. Is there a specific reason why the authors did not characterize this interesting mutant in the context of full-length CENP-E? Since the acidic patch mutant prevents binding to BUBR1, it would be a precise tool to assess the functional significance of this interaction in cells and would have nicely complemented the BubR1(1-1030) mutant in Figure 4. Related to this, the authors conclude on page 10: "On CENP-E a small acidic patch is critical to specify the interaction with BubR1. Mutation of these amino acids prevents the targeting of this CENP-E(2055-2358) domain to kinetochores and consequently compromises chromosome alignment." The conclusion about chromosome alignment is not valid because this would have to be assessed in the context of full-length mutated CENP-E.
We agree with the reviewer. That would have been an excellent experiment to make full-length CENPE 4E mutant to test its function in cells and we tried in multiple ways to generate this construct. However the plasmid is so large we failed to generate the mutant. We believe the BubR1 1-1030 is a good mutant to specifically disrupt the interaction with CENP-E and that in the light of this experiment, the FL CENPE mutant would be redundant. We have also removed the "and consequently compromises chromosome alignment"

- As shown in Figure 4, GFP::BubR1(1-1030) results in misaligned chromosomes with high levels of CENP-E. The authors conclude that "The BubR1-dependent recruitment of CENP-E to kinetochores is therefore essential for correct biorientation of kinetochores." (page 10). Why does it have to be related to recruitment per se? I am not disputing the conclusion that BubR1 facilitates initial kinetochore recruitment of CENP-E, but the authors should also consider the possibility that the effect of the GFP::BubR1(1-1030) mutant on chromosome congression may reflect a role of the BubR1 interaction in regulating CENP-E activity.

We agree and have now included: BubR1 may also regulate CENP-E activity at kinetochores.

Other minor issues/suggestions:

- Figures in general: in many instances (e.g. Figure 1D and F), the CENP-E residue numbers are too small to read comfortably. I would recommend not using subscript for residue numbers or increasing font size.
We have now made the font larger.

- Figure 1D: it would be good to show some blow ups of the polar kinetochores for GFP::CENP-E(2055-2608).
We have now provided blow ups of kinetochores, using ACA as a kinetochore marker.

- Figure 2C: it might be informative to include additional Bub1 orthologs in the aligment to show the extent to which this region is divergent between BubR1 and Bub1.
We have included this in Fig 2C.

- Figure 4: it would be helpful to indicate in the figure panels that BubR1 is depleted by RNAi.
We have now added this information to the figure.

- Figure S3B: in addition to the number of peptides the % sequence coverage should also be shown.
We have now included this.

- Page 3: "Kinetochore-bound CENP-E moves laterally attached chromosomes to the cell equator along microtubules (Wood et al., 1997). It would be appropriate to also cite Kapoor et al (2006), PMID 16424343.
We agree and have now included this reference.

- Page 6, repetitive sentence: "To stabilize it while mimicking dimeric CENP-E(2055-2608), we fused it to a C-terminal GST and removed 14 residues at the N terminus, to stabilize it while mimicking dimeric CENP-E(2055-2608)."

© 2020. Published by The Company of Biologists under the terms of the Creative Commons Attribution License (https://creativecommons.org/licenses/by/4.0/).

We apologize for this mistake and have removed this repeated clause.

- Page 7: “We found a longer loop in the C terminus of BubR1…”, but then later “on its own this basic helix in BubR1…”. Is it a loop or a helix?
We thank the reviewer for spotting this mistake and apologize and have replace loop by predicted helix.

- Page 9: “After a longer nocodazole treatment (60 minutes),…(Fig 4G-I)”. In Figure 4, the nocodazole treatment is said to be 2.5 h.
We apologize for this mistake and have edited the text.

Reviewer 2 Comments for the Author:
Major points:

1.What was the control for the MS analysis? How did the authors ensure that the interaction between CENP-E 2055-2358 and BubR1 or MYRT1 is specific? Can we be sure that this interaction occurs at kinetochores? CENP-E 2055-2358 appears to also bind CEP170 (17 peptides), Cep55 (14 peptides) and Aurora-A (12 peptides). One would expect these interactions to occur at centrosomes, where the CENP-E 2055-2358 fragment does not bind (Fig. 1 B and C).
Our control for the mass spec analysis was beads only. In the CENP-E sample, we incubated recombinant his-CENP-E2055-2358 to beads. We have provided more details in the methods section of how the experiment was performed. This experiment was the basis for the study and validation in this manuscript of the BubR1-CENP-E interaction. Indeed we show by reconstituting the interaction in vitro and we show it is specific at the molecular level. As for MYPT1, we mentioned in the paper this protein was an interesting candidate, as it is at kinetochores but not further studied in this manuscript. We have changed our sentence from specifically interact to co-purify. Other proteins are not found at kinetochores so we have not mentioned them in the manuscript and are not further studied here.

2.Fig. 2E - The text should specify that this fragment was fused with an MBP tag that is significantly larger than 20aa of BubR1. A proper control for this experiment would be the MBP-BubR1-705-1050 fragment, which should co-elute with the CENP-E 2055-2356 fragment. Such a control would exclude the possibility that the MBP tag perturbs the interaction. Otherwise it is difficult to conclude that this basic helix region is "not sufficient" for binding to CENP-E.
We don’t believe the MBP is a problem in the assay. MBP is 42kD, slightly bigger than a kinase domain (approx 30kD). We fused the MBP at the N terminus of the BubR1-1031-1050 fragment, so that the fragment is positioned similarly to if it were fused to the kinase domain. In addition, we choose MBP specifically because it is not able to dimerize (in contrast to GST for example). Additionally, we believe that an MBP-BubR1$_{705-1050}$ construct would not necessarily be an appropriate control as the tag would be too far from the C terminal helix and is therefore very unlikely to interfere with the interaction.

3.“We then tested whether CENP-E2091-2358-GST-GFP could target to kinetochores in cells and whether this recruitment was only dependent on BubR1” (p. 7) - It will be helpful to motivate the use of the 2091-2356 fragment and not the 2055-2356 in this experiment. The 2091-2356 fragment was used specifically to assess thermodynamics of BubR1/CENP-E interaction (Fig. 2B) but was otherwise not tested in any other assay. Why not use the 2055-2356 fragment that the authors showed to bind to BubR1 in vivo (Table S1 of MS analysis) and in vitro (Fig. 2A)? Does adding GST require elimination of 22 residues from the N-terminus for in vivo kinetochore localization? If yes, this should be noted in the text.
CENP-E$_{2091-2358}$ fused to GST was generated after rounds of construct optimization for protein stability to perform ITC and structural studies, as we explained in the text. Other constructs were not stable enough to perform these experiments. Given it was the shortest construct supporting the interaction with BubR1, we further used it to define the molecular requirements for the CENPE/BubR1 interactions in vivo. Although CENP-E$_{2055-2358}$ was used to carry out the pull-down and initial biochemical experiments, removing some N terminal amino acids greatly improved the stability of this construct while conserving the interaction with BubR1. This was essential to perform the ITC experiment, where degradation prevented data analysis. However we believe it is

© 2020. Published by The Company of Biologists under the terms of the Creative Commons Attribution License (https://creativecommons.org/licenses/by/4.0/).

unlikely that the GST fusion, which is C-terminal would require N-terminal truncation to localize to kinetochores, especially that the acidic patch is at the C terminus of the construct.

4.“These data indicate that while CENP-E can localize to the outer corona, the RZZ complex is not a CENP-E recruiting-factor to kinetochores, as shown previously (Pereira et al., 2018)” (p. 8) - As the authors stated, the fibrous corona is most prominent on unattached kinetochores. Therefore, it is not surprising that depletion of corona components did not change CENP-E levels at attached kinetochores. This does not exclude that corona components could contribute to the prometaphase CENP-E recruitment. To conclude that RZZ complex does not contribute to CENP-E recruitment, the authors should show that CENP-E levels are still high at unattached kinetochores under ZW10 depletion. It is also not clear from the context if the authors corroborate or contradict previous findings by Pereira et al., 2018.
We have now clarified the text. We agree that we have not tested that the RZZ complex contributes or not to CENP-E recruitment.

5.Fig. 4A-I. - One would expect that unattached kinetochores harbored more CENP-E, but the quantitation does not show an increase in the CENP-E/CENP-C ratio. If the ratios were normalized relative to WT in each experiment, then this should be explained in the legend. Along these lines CENP-E levels should go up at unattached kinetochores created by brief nocodazole treatment. Therefore, it is likely that CENP-E levels went up in both BubR1 WT and "no induction" (due to residual BubR1 left), while the BubR1 1-1030 fragment failed to recruit CENP-E to the newly formed unattached kinetochores. This is consistent with the authors’ idea that BubR1 is important for initial CENP-E recruitment. However, with 2.5 h nocodazole treatment, it looks like CENP-E levels are higher with the 1030 fragment compared to no induction. The authors stated that CENP-E levels are equal in these conditions, but the statistics represented in Fig. 4H do not test these two conditions. In fact, it appears that BubR1 1-1030 expressing cells are more similar to BubR1 WT than to “no induction”. This result seems inconsistent with the conclusions that BubR1 “is not strictly required for CENP-E localization” (p. 9) and that “another pathway also promotes CENP-E localization to kinetochore in the absence of BubR1” (p. 9).
We updated the legends to make it clear that the CENP-E/CENP-C ratios were normalized to WT. In the situation where cells had been treated with nocodazole for 2.5 h the differences between no BubR1, BubR1 WT and BubR1 1-1030 are small but statistically significant, consistent with the idea that even in this situation BubR1 contributes to the total pool of CENP-E at kinetochores, albeit to a much reduced extent. This has now been explained more clearly in the text.

6.Fig, 4A-I - The figure reads 2.5h = 150 minutes nocodazole, not 60 minutes as described in the text. Was nocodazole added together with MG132? If yes, then one would assume that these kinetochores were likely never attached unlike the kinetochores measured in Fig. A-C. This might contribute to the different phenotype observed in Fig. 4D-F and Fig. 4G-I, since kinetochore composition and biochemical state change throughout mitosis. While nocodazole treated cells lose attachments and recruit SAC components, it is unclear if artificial destabilization of fully mature attachments (MG132) can be compared with initial CENP-E recruitment at kinetochores that have never been attached. One example of this is PP1 phosphatase that lingers at kinetochores even after 30min of high nocodazole treatment (DeLuca et al., 2011 J Cell Sci). Also, what happens to the fibrous corona that was most likely gone in MG132 treated cells? Is it reformed? A better way to assess the requirement of BubR1 for initial CENP-E recruitment would be to quantify CENP-E in prometaphase cells when the initial attachments take place. Unless these points can be addressed, the statement that the “BubR1 facilitates initial CENP-E recruitment to SAC-active kinetochores” (p.9) should be toned down.
Thank you for pointing out the discrepancy for the length of the nocodazole treatment, this has now been corrected. While we agree that MG132 treatment followed by a short nocodazole treatment is not identical to the early recruitment of kinetochore components that is naturally observed at the entry of mitosis, our synchronization protocol allows us to analyse a large number of cells under precisely defined conditions. We have acknowledged the referee’s concerns by changing the statement “BubR1 facilitates initial CENP-E recruitment to SAC-active kinetochores” to “Taken together, these results suggest that BubR1 primarily facilitates initial CENP-E recruitment to SAC-active kinetochores…”

© 2020. Published by The Company of Biologists under the terms of the Creative Commons Attribution License (https://creativecommons.org/licenses/by/4.0/).

7.Fig. 4L - There are multiple bands on the gel in the "induced" lanes. Are these degradation products? Since these are well-defined bands, they may indicate stable truncations fragments bearing BubR1 epitope. The authors should indicate which bands correspond to the expected BubR1 protein/fragment. Also, one can see a faint band of BubR1 size in BubR1 1-1030 and BubR1 WT cells under BubR1 depletion. It is then possible that the increased level of CENP-E in BubR1 1-1030 mutant as compared to "No induction" condition (Fig. 4A-B) is due to either higher level of the endogenous BubR1 or to the presence of some truncation products. A blot for GFP could help show which bands are the induced proteins as opposed to endogenous BubR1.
We agree probing for GFP would be a useful experiment, however before the lockdown, we had problems with the GFP antibody not working. We have now clearly labeled the different BubR1 species on the Western blot in Figure 4L. The bands pointed out by the referee are indeed degradation products.

8.“In the absence of this CENP-E pool at kinetochores, the kinetochore-microtubule attachment is compromised, even when high levels of CENP-E are present (Fig 4J, K)” (p. 10). - How did the authors define "high levels" of CENP-E in Fig. 4J? It is possible that the few unaligned chromosomes in BubR1 1-1030 harbor more CENP-E than "no induction" cells, but less CENP-E than prometaphase BubR1 WT cells prior to biorientation. This comparison is needed to justify the phrase “even when high levels of CENP-E are present".
We have changed the text to explain our observations more clearly. The text now reads: “In the absence of this BubR1-associated pool of CENP-E at kinetochores the kinetochore-microtubule attachment is compromised, even when other, not BubR1-associated, CENP-E molecules are present (Fig 4J, K).”

Minor points:

1. Introduction - It would be useful for the readership to cite the recent experimental evidence that kinetochore expansion maximizes capture via fibrous corona (Sacristan et al., 2018 NCB).
We have now included that reference.

2. Introduction p.2 – “CENP-E is enriched at unattached and misaligned kinetochores in early mitosis”. Although this may be a common observation in the mitotic field, it is important for the broader cell biology readership to cite previous work showing this.
We have now included a reference to a review on CENP-E.

3. Introduction - Was centrosome localization of the endogenous CENP-E reported previously? If yes, a citation of the relevant work is needed here.
We have now included a citation to Maffini et al 2009.

4. “Indeed full-length BubR1 interacts with CENP-E2055-2608 in vitro (data not shown).” (p.4) – Why is this data not shown?
We have many gel filtration profiles in the paper and showing that the pseudokinase domain interacts with CENP-E2055-2608 is more precise than with full length BubR1. We also have gel filtration profiles for BubR1/Bub3 and Bub1/Bub3 with CENP-E2055-2608 showing only BubR1/Bub3 co-elutes with CENPE. However we feel it does not really add anything to the paper and adding all these gel filtrations profiles would result in the important information getting diluted. We have now modified the text to say we have done the gel filtration with BubR1/Bub3/CENPE and quote Ciossani et al, who also did that in their paper.

5. Fig. 1B and C - What was the reason for looking at CENP-E localization in metaphase? The primary function of CENP-E is chromosome congression and its highest kinetochore localization is observed in early mitosis, therefore one might miss kinetochore-localization domains used prior to congression. As the authors pointed out, CENP-E localization changes throughout mitosis, presumably due to the composition of the fibrous corona and SAC proteins. Therefore, it would be informative to compare the behavior of given fragment in early vs late mitosis. A protein fragment in its localization at kinetochores throughout mitosis would be a great candidate for a SAC-independent localization.

© 2020. Published by The Company of Biologists under the terms of the Creative Commons Attribution License (https://creativecommons.org/licenses/by/4.0/).

We thank the reviewer for this excellent point and we will examine this when the labs open again and people have childcare and can go back to work. Mainly we examined the constructs by transient transfections, after failing to make stable cell lines of the constructs because some of them are dominant negative. Most cells we could observe were in metaphase and so we decided to examine CENP-E localization mainly in metaphase for comparison. At the moment, we do not have images of prometaphase-transfected cells for each construct, preventing this thorough analysis. However we are excited to revisit this when possible. In the meantime, the localization of the CENP-E constructs at metaphase stage provide useful information about the constructs and their targeting.

6. Fig. 1B and C - 2260-2608 fragment is not depicted.
This fragment is in Figure S1C. We have now included this information in the figure legend.

7. Fig. S1D and E – " However, GFP-CENP-E2055-2608 was strongly enriched at kinetochores close to spindle poles, suggesting GFP-CENP-E2055-2608 was recruited to these kinetochore subpopulations independently of endogenous CENP-E, through another binding partner (Fig 1D, S1E)" (p. 2). Bringing both graphs to the same scale would help assess this statement. Currently the median value for polar chromosomes is unreadable. Is there a significant difference between conditions tested in S1E graph? If so, suggesting that GFP-CENP-E recruitment at polar chromosomes is independent on the endogenous protein, is most likely incorrect.
The data is not normally distributed and does not follow a Gaussian curve. Therefore displaying the mean would not be appropriate. We have now brought the graphs to the same scale. There is no statistical difference in S1E and we have now edited this.

8. "Overall these results indicate CENP-E2055-2608 targeting to kinetochores outcompetes endogenous CENP-E" (p. 2). - "Outcompetes" implies some kind of direct measurement of competition between the endogenous and exogenous CENP-E. However, we do not know the ratio of these proteins in the cytoplasm. Based solely on decreased immunofluorescent signal, it would be safer to simply say "... competes with endogenous CENP-E".
We have now change this, as suggested.

9. "To stabilize it while mimicking dimeric CENP-E2055-2608, we fused it to a C- terminal GST and removed 14 residues at the N terminus, to stabilize it while mimicking dimeric CENP-E2055-2608" (p. 5) – there is a repetition in this sentence.
We apologize for this mistake and have removed this repeated clause.

10. Table S1 - One of the highest scoring proteins is CENP-E itself. Did the authors detect any peptides belonging to the endogenous CENP-E? Absence of such peptides would reinforce the finding that this protein region lacks dimerization capacity in vivo.
We have now described the methods for this experiment in more details. We incubated NiNTA beads with recombinant protein, which was highly enriched in comparison to the cytoplasmic extracts. Therefore while we could identify some proteins bound by mass spectrometry, the data are not quantitative and because CENPE2055-2358 was the bait in high abundance (hence many peptides recovered), it is unlikely we can determine the domain would not dimerize in vivo.

11. Isothermal titration calorimetry (ITC) - This paragraph would benefit from additional explanation (method section). How was the stoichiometry determined to be 1:1 and what led to the conclusion that CENP-E motor is able to bind two molecules of BubR1?
We fitted the ITC data to a one to one binding model as already quoted in the method section. We fix the concentrations of the proteins and float the number of binding sites (N) of the protein in the cell (CENPE), the Kd and the enthalpy of the interaction, deltaH. This converges with N = 0.908 +/- 0.016 very close to a stoichiometry of 1:1. We have included more details in the method section and included the number of measured binding sites in the result section.

12. "We then examined the sequence conservation between Bub1 and BubR1 kinase domains." - It is not clear from the text why authors are performing such comparison. Bub1 peptides (3) were found in the MS analysis using CENP-E 2055-2356 fragment (Table S1). Therefore, why would one expect that the longer loop in the C-terminus of BubR1 absent in Bub1 be responsible for specific CENP-E 2055-2356 and BubR1 interaction? This line of reasoning requires additional explanation.

© 2020. Published by The Company of Biologists under the terms of the Creative Commons Attribution License (https://creativecommons.org/licenses/by/4.0/).

The Bub1 peptides were only found in the control sample (beads only), but not bound to CENP-E 2055-2356. We have also included" Previous work highlighted that the C terminus of Bub1 recruits CENP-F to kinetochores (Raaijmakers et al, 2018)", which also enabled us to hypothesize that the C terminus of BubR1 recruits CENP-E in a similar fashion.

13. "Indeed, CENP-E2069-2358-GST did not co-elute with BubR1705-1030 lacking…" – Do the authors mean 2055-2356 fragment? As a general comment, the authors should ensure that all the fragment names in the text match the names in the figures.
As explained in the text, the constructs CENP-E2069-2358 and CENP-E2091-2358, designed later were increasingly more stable and the later experiments were performed with these constructs. It would have been clearer to perform all the experiments with these constructs rather than CENP-E2069-2358 at the start. However the current situation prevents such repeat experiments and they would not contribute any novel information to the paper.

14. "Since BubR1 mediates the kinetochore localization of only one pool of CENP-E (see below), this suggests…" (p. 8) - It is challenging to follow the reasoning based on the result that was not yet presented. Alternatively the authors could specify which figure are they referring to when saying "below". Up to this moment in the text, the authors did not provide any evidence that BubR1 mediates recruitment of any CENP-E pool to kinetochores. We only know that BubR1 and CENP-E interact, possibly at kinetochores (see Major comment 1).
We have removed this sentence as we mention in the text later the existence of an alternate pathway.

15. Fig. S3C - What was the reason for not using the CENP-E 2055-2358 fragment that is sufficient to bind BubR1 according to the MS analysis?
We initially designed and worked with the CENP-E kinetochore targeting domain, reported to be 2055-2608 (Chan et al, 1998). We performed these interaction/mass spectrometry studies as a starting point to identify CENP-E kinetochore interacting partners in a non-biased approach using mitotic extracts. However, the point of the study was defining the molecular basis of interactions between CENP-E and partners. Unfortunately, we did not obtain diffraction-quality crystals of CENP-E. We also performed negative stained EM on CENP-E (2055-2608) bound to BubR1/Bub3. The sample on grids was not homogenous and fell apart on the grids, thus we could not obtain class averages.

16. "To evaluate BubR1's contribution to CENP-E localization (…) Fig. 4A, S4A" (p.9) – Figure S5A should be referred to here.
We have now included it.

17. Fig. 4A-K – Images and graphs should be annotated as "siBubR1" in all conditions. This would make it easier to read the figure without digging into the figure legend.
We have now edited the figure.

18. Fig. 4 C, F, I - were the CENP-E levels compared to BubR1-GFP signal or to the total BubR1? It is important to make this distinction because siRNA never depletes 100% of the endogenous protein.
CENP-E levels were compared to GFP-BubR1. This is now explained in the legends.

19. Consistent with the minor comment #20 – Why are CENP-E/ACA intensity ratios in Fig. 1S reported as medians but CENP-E/CENP-C ratios in Fig. 4 as means?
See point 7.

20. Fig. S4A - The authors showed that CENP-E 2055-2608 from bacteria and BubR1 432-1050 from insect cells interact in vitro. What is the reason behind using a different BubR1 fragment (705-1050) in the analysis represented in Fig. S4A? This fragment lacks 273aa as compared to the previously tested BubR1 432-1050.

We demonstrated first that CENP-E 2055-2608 bind to the C terminus of CENPE but not the N terminus (Fig S3). This experiment in Fig S3 establishes what domain of BubR1 interacts with CENPE. However while we were doing these experiments, the Musacchio lab published BubR1 705-1050 binds to CENPE in vitro. For reductionist biochemical studies and identify essential

© 2020. Published by The Company of Biologists under the terms of the Creative Commons Attribution License (https://creativecommons.org/licenses/by/4.0/).

components, it is better to use the smallest functional constructs. Thus for most other experiments done with BubR1, we used the construct 705-1050. The reference to this construct is listed in the text on page 6.

21. Fig. S5A - It should be explicitly mentioned in the figure legend that only metaphase plate kinetochores were counted, since Bub1 depletion generates some unaligned kinetochores.
We have included this statement in the legends.

22. Methods section, Microscopy - There is no mentioning of Aurora-B or Aurora-A inhibition in this paper but the names and concentrations of their inhibitors (ZM1 and MLN) are listed.
We have now removed them.

Second decision letter

MS ID#: JOCES/2020/246025

MS TITLE: The C-terminal helix of BubR1 is essential for CENP-E-dependent chromosome alignment

AUTHORS: Thibault Legal, Daniel Hayward, Agata Gluszek-Kustusz, Elizabeth Blackburn, Christos Spanos, Juri Rappsilber, Ulrike Gruneberg, and Julie Welburn
ARTICLE TYPE: Research Article

We have now reached a decision on the above manuscript.

To see the reviewers' reports and a copy of this decision letter, please go to: https://submit-jcs.biologists.org and click on the 'Manuscripts with Decisions' queue in the Author Area. (Corresponding author only has access to reviews.)

As you will see, the reviewers gave favourable reports but raised some critical points that will require amendments to your manuscript. I hope that you will be able to carry these out, because I would like to be able to accept your paper.

We are aware that you may currently be unable to access the lab to undertake experimental revisions. If it would be helpful, we encourage you to contact us to discuss your revision in greater detail. Please send us a point-by-point response indicating where you are able to address concerns raised (either experimentally or by changes to the text) and where you will not be able to do so within the normal timeframe of a revision. We will then provide further guidance. Please also note that we are happy to extend revision timeframes as necessary.

Please ensure that you clearly highlight all changes made in the revised manuscript. Please avoid using 'Tracked changes' in Word files as these are lost in PDF conversion.

I should be grateful if you would also provide a point-by-point response detailing how you have dealt with the points raised by the reviewers in the 'Response to Reviewers' box. Please attend to all of the reviewers' comments. If you do not agree with any of their criticisms or suggestions please explain clearly why this is so.

Reviewer 1

Advance summary and potential significance to field

See review of original submission.

© 2020. Published by The Company of Biologists under the terms of the Creative Commons Attribution License (https://creativecommons.org/licenses/by/4.0/).

Comments for the author

The authors responded satisfactorily to all of my comments and made changes to the manuscript text and figures where appropriate. I have no further concerns and recommend publication.

Reviewer 2

Advance summary and potential significance to field

See previous review

Comments for the author

The manuscript is improved by including explanations for most of the concerns raised in the original review. One remaining point should still be addressed.

Major point 2 from the original comments:
Fig. 2E - The text should specify that this fragment was fused with an MBP tag that is significantly larger than 20aa of BubR1. A proper control for this experiment would be the MBP-BubR1-705-1050 fragment, which should co-elute with the CENP-E 2055-2356 fragment. Such a control would exclude the possibility that the MBP tag perturbs the interaction. Otherwise it is difficult to conclude that this basic helix region is "not sufficient" for binding to CENP-E.

Authors' response:
We don't believe the MBP is a problem in the assay. MBP is 42kD, slightly bigger than a kinase domain (approx 30kD). We fused the MBP at the N terminus of the BubR1-1031-1050 fragment, so that the fragment is positioned similarly to if it were fused to the kinase domain. In addition, we choose MBP specifically because it is not able to dimerize (in contrast to GST for example). Additionally, we believe that an MBP-BubR1705-1050 construct would not necessarily be an appropriate control as the tag would be too far from the C terminal helix and is therefore very unlikely to interfere with the interaction.

New comment:
The issue is that there is no positive control for the experiment (Figure 4E in the revised manuscript). The data show no interaction between MBP-BubR1-1031-1050 and CENP-E-2069-2358, and the authors' interpretation is that this 20 aa fragment of BubR1 is insufficient for binding. However, in the absence of any data showing that another MBP-tagged fragment can interact with CENP-E (i.e., a positive control), another interpretation is that the MBP tag interferes with binding. Without a positive control, the interpretation should be more cautious and point out the difference between this assay and others in the manuscript without MBP tags.

Second revision

Author response to reviewers' comments

The issue is that there is no positive control for the experiment (Figure 4E in the revised manuscript). The data show no interaction between MBP-BubR1-1031-1050 and CENP-E-2069-2358, and the authors' interpretation is that this 20 aa fragment of BubR1 is insufficient for binding. However, in the absence of any data showing that another MBP-tagged fragment can interact with CENP-E (i.e., a positive control), another interpretation is that the MBP tag interferes with binding. Without a positive control, the interpretation should be more cautious and point out the difference between this assay and others in the manuscript without MBP tags.

We have now modified the text to the following:

© 2020. Published by The Company of Biologists under the terms of the Creative Commons Attribution License (https://creativecommons.org/licenses/by/4.0/).

However, on its own this basic helix in BubR11031-1050 (pI=10.30) fused to MBP, was not sufficient to interact with CENP-E (Fig 4E), although we cannot rule out that the MBP would disrupt the interaction.

Third decision letter

MS ID#: JOCES/2020/246025

MS TITLE: The C-terminal helix of BubR1 is essential for CENP-E-dependent chromosome alignment

AUTHORS: Thibault Legal, Daniel Hayward, Agata Gluszek-Kustusz, Elizabeth Blackburn, Christos Spanos, Juri Rappsilber, Ulrike Gruneberg, and Julie Welburn
ARTICLE TYPE: Research Article

I am happy to tell you that your manuscript has been accepted for publication in Journal of Cell Science, pending standard ethics checks.

© 2020. Published by The Company of Biologists under the terms of the Creative Commons Attribution License (https://creativecommons.org/licenses/by/4.0/).
